# Wuling capsule alleviates hyperuricaemia and protects UA- injured HK-2 cells by regulating uric acid transporter proteins

**DOI:** 10.3389/fphar.2025.1563676

**Published:** 2025-04-29

**Authors:** Nan Li, Hongna Liu, Zhongxing Song, Rui Zhou, Zhishu Tang, Hongbo Xu, Xinbo Shi, Yanru Liu, Jian Ni

**Affiliations:** ^1^ State Key Laboratory of Research and Development of Characteristic Qin Medicine Resources (Cultivation), Co-Construction Collaborative Innovation Center for Chinese Medicine Resources Industrialization by Shaanxi and Education Ministry, Shaanxi Innovative Drug Research Center, Shaanxi University of Chinese Medicine, Xianyang, China; ^2^ Tsing Hua De Ren Xi’an Happiness Pharmaceutical Co., Ltd., Xi’an, China; ^3^ School of Traditional Chinese Medicine, Beijing University of Chinese Medicine, Beijing, China

**Keywords:** Wuling capsule, hyperuricaemia, urate transporter, HK-2 cells, apoptosis

## Abstract

**Introduction:**

Wuling capsule is a Chinese patent medicine mainly used for the treatment of chronic liver disease in clinical practice. Our previous work has revealed that Wuling capsule could inhibit liver fibrosis by regulating macrophage polarization, and firstly demonstrated its anti-gout effects on monosodium urate (MSU)- induced acute gouty arthritis (AGA) in rats. High uric acid (UA) levels are known to be the primary cause of gout. Therefore, this study investigated the UA lowering, kidney protection effects and underlying mechanisms of Wuling capsule in vivo and in vitro, and also determined its key bioactive constituents.

**Methods:**

The efficacy of Wuling capsule for HUA symptoms in rats was evaluated. Histopathological analysis of liver and kidney tissues were detected by HE staining. The biochemical indices were measured using specific kits. The main constituents of Wuling capsule and its medicated serum were analyzed by UPLC-QTOF-MS/MS. Protective effects of saikosaponin A, tanshinone IIA, schisandrol B, and ganoderic acid A on UA-injured HK-2 cells were assessed via Hoechst 33342/PI staining and flow cytometry. Molecular docking and dynamics simulation predicted the binding energy and stability of these constituents to UA related transporters. The mRNA and protein expression levels of UA related transporters were examined using RT-qPCR and Western blotting.

**Results:**

In HUA rats, Wuling capsule significantly reduced the serum UA level and xanthine oxidase (XOD) content in both serum and liver. Furthermore, it improved liver function markers (ALT, AST) and renal injury indicators (Cr, BUN), ameliorated renal tubule dilation and inflammatory infiltration in the kidney, and regulated the mRNA and protein expression of UA related transporters (URAT1, GLUT9, ABCG2 and OAT1). In vitro, the main constituents of Wuling capsule (saikosaponin A, tanshinone IIA, schisandrol B and ganoderic acid A) improved cell viability and inhibited cell apoptosis in UA-injured HK-2 cells. Subsequently, its four serum constituents also significantly regulated the mRNA and protein expression of URAT1, GLUT9, and ABCG2 selectively.

**Discussion:**

This work demonstrated the therapeutic effect of Wuling capsule on HUA by protecting liver and kidney function and regulating UA related transporters. These findings provide novel support for the further clinical application of Wuling capsule.

## 1 Introduction

Hyperuricaemia (HUA) can be diagnosed in people consuming a normal diet when fasting serum uric acid (UA) levels exceed 420 μmol/L. HUA has become the second most prevalent metabolic disease disorder after diabetes (Zhao et al., 2022). Disrupted purine metabolism leads to elevated UA levels in the body (Danve et al., 2021), which is the main cause of gout (Wen et al., 2024). Recent studies indicate that approximately 930 million individuals are affected by HUA and gout worldwide. In China, the overall prevalence of HUA is 13.3%, affecting approximately 177 million people (Zhang et al., 2021). Reducing UA production and increasing its excretion are crucial for the prevention and treatment of HUA. In clinical practice, analgesics (such as colchicine and glucocorticoids), xanthine oxidase inhibitors (such as febuxostat tablets and allopurinol tablets) and urate transporter inhibitors (probenecid and benzbromarone) are commonly used to manage of HUA and gout (Roddy et al., 2023). However, the long-term use of colchicine, glucocorticoids, allopurinol and benzbromarone could result in adverse effects such as diarrhoea, nausea, vomiting and significant hepatic or renal impairment (LaForge et al., 2023). Consequently, identifying more effective and safer pharmacological agents for the prevention and treatment of HUA is imperative.

UA is synthesized primarily in the liver and is subsequently excreted via the kidneys (Mei et al., 2022). UA is the final product of both exogenous purines derived from dietary sources and endogenous purines originating from damaged or necrotic cells. Approximately 20% of exogenous purine compounds and 80% of endogenous purine nucleotides are catalysed by phosphomonoesterase or nucleotidase to yield nucleosides, which can undergo further decomposition. This decomposition process is facilitated by two types of enzymes: nucleoside phosphorylase, which catalyses the conversion of nucleosides into nitrogenous bases and pentose 1-phosphate, and nucleoside hydrolase, which catalyses the conversion of nucleosides into nitrogenous bases and pentoses. Adenosine deaminase catalyses the deamination of adenosine monophosphate to produce hypoxanthine, which is oxidised to xanthine by xanthine oxidase and ultimately hydrolysed to UA (Toledo-Ibelles et al., 2021). Similarly, 5′-guanylic acid is converted to guanine, which is then transformed into xanthine and subsequently further converted to UA. In the UA excretion pathway, approximately 70% of UA is excreted via the kidneys. After filtration through the glomeruli, UA undergoes absorption, secretion, and reabsorption within the proximal renal tubules. Within these tubules, up to approximately 90% of the UA is reabsorbed and secreted back into the lumen of the renal tubule. This is the main cause of the high level of UA in the serum (Guo et al., 2023). Therefore, inhibiting UA production in the liver or promoting UA excretion in the kidneys represents one of the most promising therapeutic strategies currently available.

Xanthine oxidase (XOD) is a crucial enzyme in the synthesis of UA, and reducing XOD activity can effectively inhibit UA production in the liver (Furuhashi, 2020). Recent studies have shown that UA-related transporters play a significant role in the treatment of HUA. Uric acid transporter 1 (URAT1) and glucose transporter 9 (GLUT9) are the key transporters involved in the regulation of UA reabsorption. Conversely, breast cancer resistance protein (ABCG2) and anion transporter 1 (OAT1) play a crucial role in the regulation of UA excretion. Numerous studies have demonstrated that various Chinese herbal medicines can effectively lower serum UA levels without causing significant hepatic or renal toxicity (Qian et al., 2023). Current research consensus supports the notion that protecting liver and kidney function, reducing XOD activity, inhibiting UA synthesis, and promoting UA excretion are effective therapeutic strategies for HUA (Saka et al., 2021).

Previous works indicated that patients with hyperuricemia often have insufficient liver and kidney functions (Johnson et al., 2024). Therefore, in addition to lowering serum UA, promoting liver and kidney functions may be an effective strategy for curing hyperuricemia. Wuling capsule is a traditional Chinese medicine recognized in the Chinese Pharmacopoeia, which exhibits a range of pharmacological effects, including liver protection, anti-inflammatory, and immunomodulatory effects, as well as safety in widespread clinical application (Committee, 2020). Wuling Capsule is composed of four herbs: Bupleurum Radix, Salviae Miltiorrhizae Radix ET Rhizoma, Schisandra chinensis Fructus and Ganoderma. In our previous work, we demonstrated that Wuling Capsule exerts notably liver protection effects in CCl_4_-induced liver injured rats through mitigating collagen deposition by inhibiting the TLR4-NF-κB signaling pathway (Ren et al., 2024). Based on the theoretical basis of “liver and kidney homology” of traditional Chinese medicine, modern pharmacological researches put forward a new strategy of “protecting the function of liver and kidney” against gout and reducing UA (Bai et al., 2024). We hypothesise that Wuling Capsule might alleviate gout and hyperuricemia through improving liver and kidney function. Thus, we preliminarily have demonstrated that Wuling capsule exerts a significant therapeutic effect on acute gouty arthritis (AGA) in rats by suppressing the expression of inflammatory factors in both the blood and synovium (Nan et al., 2024). Given that elevated levels of UA in the blood are directly implicated in the pathogenesis of AGA and HUA, this study aimed to investigate the effects of Wuling Capsule on HUA and the underlying mechanism of its action.

## 2 Materials and methods

### 2.1 Drugs and reagents

Wuling Capsules are provided by Qinghua Deren Xi’an Xingfu Pharmaceutical Co., Ltd.; Allopurinol Tablets were purchased from Hefei Jiulian Pharmaceutical Company; Hypoxanthine and Potassium Oxonate (PO) were purchased from Shanghai Yuanye Bio-Technology Co., Ltd. in China; Uric Acid (UA), Xanthine Oxidase (XOD), Creatinine (Cr), and Blood Urea Nitrogen (BUN) assay kits were purchased from Nanjing Jiancheng Bioengineering Institute(Catalog No.C012-2-1, A002-1-1, C011-2-1, C013-2-1); ALT and AST were purchased from Shenzhen Mindray Bio-Medical Electronics Co., Ltd.(Catalog No.140121004, 140221008); UA was purchased from Sigma-Aldrich; (Catalog No.U2625). Salvianolic acid B (A0056, 98.78%), Schisandrol B (A0208, 99.88%), and Schisandrin A (A0203, 99.78%), Schisanhenol (A0823, 99.87%) were all purchased from Chengdu Manster Biotechnology Co., Ltd.; Schisandrol A (Batch No.: 110857-201815, 99.7%) and Tanshinone IIA (Batch No.: 110766-200416, 98.9%) were both purchased from the National Institutes for Food and Drug Control; Saikosaponin A (B20146, 98%) was purchased from Shanghai Yuanye Biotechnology Co., Ltd.; Acetonitrile (chromatographically pure) was purchased from Honeywell in the United States.

### 2.2 Animal

Male Sprague-Dawley rats (180 ± 20) g were procured from Chengdu Dashuo Experimental Animal Co., Ltd (License No. SCXK (CHUAN2020-030). The experimental protocols involving these animals received approval from animal Experimental Ethics Committee of Shaanxi University of Chinese Medicine (Ethics Number: SUCMDL20230614001; Date: June. 14, 2023). The rats were maintained in a barrier system under controlled conditions, including a standard 12 h light-dark cycle, a temperature of 20 ± 2°C, and a relative humidity of 45 ∼ 70%.

### 2.3 HUA rat model establishment and treatments

The established clinical dosage of the Wuling capsule for human administration is 5.25 g per day. In animal studies, dosage conversion was performed based on body surface area calculations. For rats, the dosage was determined by using the formula: 5.25 g/70 kg × 6.3 = 0.4725 g/kg. Consequently, a medium dosage of 0.5 g/kg was established for the Wuling capsule, with 0.25 g/kg designated as the low dosage and 1 g/kg as the high dosage. The dosage of hypoxanthine and PO were selected according to published literature (Bao et al., 2022; Jing et al., 2021).The HUA rat model was established using hypoxanthine in combination with potassium oxonate. In brief, a total of 72 rats were randomly assigned to 6 groups, each comprising 12 rats: a control group, a model group, three treatment groups receiving Wuling capsules (0.25 g/kg, 0.5 g/kg, or 1 g/kg), and a treatment group receiving allopurinol (27 mg/kg). With the exception of the control and model groups, allopurinol and varying doses of Wuling capsule were administered intragastrically over a period of 15 days. On Day 8, all groups, with the exception of the control group, were administered 500 mg/kg hypoxanthine and were injected with 100 mg/kg potassium oxonate (PO) for 8 days to establish a HUA rat model. Following a 15 days period, the rats were subjected to abdominal anaesthesia with 2.0% pentobarbital sodium, after which blood samples were collected for subsequent serum biochemical analysis. The liver, spleen and kidney were excised and weighed. Liver and kidney samples from each group were subjected to histopathological examination and biochemical analysis.

### 2.4 Preparation of drug-containing rat sera

After a one-week adjustment period, the rats were randomly allocated to 2 groups (n = 8 per group): the normal serum group (rats received an equivalent volume of pure water) and Wuling capsule serum group (rats received Wuling capsule at a dosage of 5 g/kg) once daily. The freeze-dried powder of Wuling capsule was dissolved in pure water and administered intragastrically from Day 1 to Day 7. Two hours after the last dose, the rats were anaesthetized with 2.0% pentobarbital sodium in advance, and rat serum was collected and stored at −80°C for future analysis.

### 2.5 Determination of body weight, liver index, spleen index and kidney index

The body weight of the rats was continuously monitored throughout the experiment with measurements recorded every 3 days. The weights of the liver, spleen and kidney of rats in each experimental group were recorded, and corresponding organ indexes were calculated using the formula. Organ index = Organ Weight (g)/Body Weight (g) *100%.

### 2.6 Biochemical analysis of serum indices and determination of liver XOD inhibitory activity

According to the manufacturer of commercial kit (Jiancheng Technology Co., Ltd., Nanjing, China), The absorbance of uric acid (UA), creatinine (Cr), blood urea nitrogen (BUN) and xanthine oxidase (XOD) in the serum or liver homogenate of the rats was measured at a wavelength of 450 nm using an enzymatic analyzer (Thermo Fisher Scientific, Waltham, MA, United States), The concentrations of these substances were subsequently determined using the specified formula. The serum levels of alanine aminotransferase (ALT) and aspartate aminotransferase (AST) in rats were measured using an automated biochemical analyzer (Shenzhen Mindray Bio-Medical Electronics Co., Ltd., Shenzhen, China).

### 2.7 Histopathological examination

Liver and kidney tissue were fixed in 4% paraformaldehyde solution for 24 h, decalcified with a 10% dilute nitric acid solution for 3 weeks, and subsequently sectioned into 5 μm slices following conventional paraffin embedding. These sections were stained with hematoxylin-eosin (HE).

### 2.8 UPLC-QTOF-MS/MS analysis

Schisanhenol, Schisandrin A, Schisandrol A, Schisandrol B, Salvianolic acid B, Tanshinone IIA and Saikosaponin A were dissolved in 80% methanol at concentrations of 1.010, 1.210, 1.190, 1.080, 1.270, 1.070 and 1.600 mg/mL respectively to prepare the standard solution. A 1 g sample of Wuling capsule was precisely weighed, and subjected to ultrasonic extraction using 25 mL of 80% methanol for 30 min. The resulting original liquid sample of Wuling capsule was then filtered through a 0.22 μm porous membrane filtration. Furthermore, 200 µL drug-containing rat sera samples mixed with 600 µL of extraction solvent (water: methanol = 20:80), The mixture was vortexed for 15 s and subsequently refrigerated at 4°C for 4 h, following this, the samples were centrifuged at 12000 rpm for 10 min at 4°C, and then dried at a low temperature. To the dried sample, 100 µL of mobile phase solution (acetonitrile: water = 1:1) was added followed by vortexing for 1 min, The mixture was centrifuged again at 12,000 rpm for 10 min at 4°C. The supernatant was carefully transferred into an internal intubation tube for sample injection. The chromatographic analysis was conducted using a Waters ACQUITY UPLC H-Class ultra-high performance liquid chromatography system, coupled with a Triple TOFTM 5600 quadrupole time-of-flight mass spectrometer (Waters, United States). Chromatography was carried out on a Waters ACQUITY UPLC HSS T3 column (2.1 mm × 100 mm, 1.8 µm particle size). The mobile phase consisted of pure water (solvent A)- and acetonitrile (solvent B). The elution conditions were: 0–3 min, 3% B; 3–20 min, 3%–37% B; 20–30 min, 37%–44% B; 30–36 min, 44%–51% B; 36–41 min, 51%–58% B; 41–49 min, 58%–68% B; 49–53 min, 68%–95% B; 53–56 min, 95% B; 56–60 min, 95%–3% B. The column temperature was maintained at 30°C, with a flow rate of 0.3 mL/min. A sample volume of 10 μL was injected. The ionization was performed using an electrospray ionization (ESI) source, and data acquisition was executed in both positive and negative ion modes. The source injection voltage (IVF) was set at +5,500 V and −4,500 V, while the cleavage voltage (DP) was maintained at ±80 V. The collision energy (CE) was adjusted to ±10 eV. Nitrogen was utilized as both the atomization gas (GS1) and the auxiliary gas (GS2), each at a pressure of 344.74 kPa (50 psi). The air curtain gas (CUR) was set at 241.32 kPa (35 psi), and the atomization temperature was maintained at 500°C. Data acquisition was performed using information-dependent acquisition (IDA), dynamic background subtraction (DBS), and high sensitivity modes. The scanning range for the parent ion (TOF-MS) was m/z 100-2,000, and the scanning range for the daughter ion was also m/z 100-2,000.

### 2.9 Cell culture

The immortalized human renal proximal tubular epithelial cell line HK-2 (HK-2). retains various functional features of the proximal tubule epithelial cells of the renal cortex, which was obtained from Wuhan Punosai Biotechnology Co., Ltd., (Wuhan, China). HK-2 cells were cultured in a special HK-2 cell medium supplemented with 10% fetal bovine serum, 1% penicillin and streptomycin mixture, and a Minimum Essential Medium (MEM) containing NEAA. (Wuhan, China). The cells were cultured in 5% CO_2_ incubators at 37°C.

### 2.10 Cell viability assay

HK-2 cells were seeded onto 96-well plates at a concentration of 1 × 10^5^ cells/mL and incubated for 12 h to facilitate adherence to the substrate. The eight constituents (Salvianolic acid B, Schisandrol B, Schisandrin A, Schisanhenol, Schisandrol A, Tanshinone IIA, Saikosaponin A, and Ganoderic acid A) with different concentration were added to HK-2 cells for 24 h incubation, to see the effect of the eight constituents on the cell viability of HK-2 cells. Then, the eight constituents with appropriate concentration were introduced into the medium for 2 h, UA serving as a stimulating agent was added to the cells. After a 24-h co-treatment, 10 µL of CCK-8 solution was added to each well, and the cells were incubated for an additional 2 h. Absorbance was measured at 450 nm using a Thermo Multiskan Sky spectrophotometer (Thermo Fisher Scientific, Waltham, MA, United States) and cellular viability was assessed.

### 2.11 Cell apoptosis assay

HK-2 cells were seeded into 6-well plates at a density of 1 × 10^5^ cells/mL. Following cell adhesion, Saikosaponin A, Tanshinone IIA, Schisandrol B, and Ganoderic acid A were administered for a duration of 2 h. And then add UA co-treatment for 24 h. Subsequently, the cells were washed with PBS, and a staining solution containing 1 mL of buffer, 5 μL of Hoechst 33342, and 5 μL of propidium iodide (PI) (Solarbio, Beijing) were added. The cells were incubated at 4°C for 20 min in the absence of light. Fluorescence microscopy was employed to observe the cells in each experimental group (Olympus, IX73). These images were obtained for further analysis. In addition, the cells were harvested, washed with PBS and stained using annexin V and PI in accordance with the annexin V-FITC apoptosis detection kit protocol (Absia, Shanghai, China). Flow cytometry and FlowJo software were employed for cell analysis.

### 2.12 Homology modelling of UA transporters

The transporters of URAT1, GLUT9, OAT1, and ABCG2 lack structural information (Chen et al., 2021). In order to further elucidate the high efficiency of four medicated serum constituents at the molecular level, we constructed the homology model of UA-related transporters. We used the Swiss model and easymode l4.0 to model homology of URAT1, GLUT9, OAT1 and ABCG2 in a multi-template mode. Search for URAT1 (accession ID: BAB96750), GLUT9 (accession ID: Q9NRM0), OAT1 (accession ID: Q4U2R8) and accession ID of ABCG2 (accession ID: Q9UNQ0) in NCBI protein Blast was used. We selected 9b1l, 8wjg, 9b1f, 9b1g and 9b1t (URAT1); 8y66, 8y65, 9CAX, 7wsn and 7wsm (GLUT9); 8bvs, 8sdu, 8bvr, 8bvt and 8bwt (OAT1); 6vxi, 6hij, 6vxf and 6vxh (ABCG2) are used as templates (Waterhouse et al., 2018). The improved model was validated with the Ramachandran diagram generated by SAVES v6.0 using UCLA-DOE LAB. (https://swissmadel.ExPASy.org/).

### 2.13 Molecular docking

The proteins URAT1, GLUT9, OAT1 and ABCG2 were employed as receptor models, whereas Saikosaponin A, Tanshinone IIA, Schisandrol B and Ganoderic acid A served as ligand molecules, representing the active constituents in the drug-containing sera of Wuling capsule. The molecules of these constituents were obtained from Traditional Chinese Medicine Systems Pharmacology Database and Analysis Platform. The structures of the UA-related transporters (URAT1, GLUT9, ABCG2 and OAT1) were modelled using homology techniques via SWISS-MODEL. Molecular docking simulations were performed utilizing the AutoDock 4.1 software. The selection of these molecular structures was based on their Goldscore fitness, and they were subsequently subjected to further evaluation through Discovery Studio 2019, which incorporates both computational assessment and visual inspection. The docking procedure was conducted as previously described, with a docking score below −4 points indicating strong binding energy (Zhou et al., 2024).

### 2.14 Molecular dynamics simulation of the main constituents of Wuling capsule

The four complexes Saikosaponin A-GLUT9, TanshinonⅡA-URAT1, Schisandrol B-OAT1 and Ganoderic acid A -ABCG2 were simulated using GROMACS (version 2022.5) software. The protein topology file is generated by the AMBER99SB-ILDN force field, and the ligand topology file is generated by the ACPYPE script using the Amber force field. Simulations of 100 ns were performed for each system under periodic boundary conditions at a temperature of 310 K and a pressure of 1.0 bar. Before the MD simulation, the steepest descent algorithm was used to minimize the energy of the treated setup, with both the Coulomb force intercept and the van der Waals radius intercept of 1.4 nm. Combined with free energy, the molecular mechanics Poisson–Boltzmann surface area (MM/PBSA) method was used for calculations and Xmgrace software 5.1.25 was used to visualize the obtained MD simulation data (Dash et al., 2021).

### 2.15 Reverse Transcription- quantitative PCR

Total RNA was isolated from rat kidney tissue and HK-2 cells using Trizol reagent (Beijing Baori Medical Technology Co., Ltd., China). The concentration of the extracted RNA was assessed using a NanoDrop One spectrophotometer. Subsequently, the total RNA was reverse transcribed into complementary DNA (cDNA). The samples were then subjected to RT-qPCR amplification using the SYBR^®^ Premix Ex Taq™ II kit (TaKaRa, Japan). The sequences of the primers utilized are provided in the Table 1, and all primers were sourced from Bioengineering Co., Ltd., Shanghai, China. GAPDH served as the endogenous control. Relative expression was quantified using the 2^−ΔΔCT^ method.

**TABLE 1:** List of prisms used in RT-qPCR for mRNA expression.

Gene	Forward primer (5′–3′)	Reverse primer (5′–3′)
Rat-GAPDH	AAA​CCC​ATC​ACC​ATC​TTC​CA	GTG​GTT​CAC​ACC​CAT​CAC​AA
Rat-URAT1	TTACGACCACAGTACCTT	ACACAGACACCAGAAGAT
Rat-GLUT9	CAA​AGA​CGA​GGA​AGC​AGT​AG	TTCATTATCGCAGGCACA
Rat-OAT1	TAA​TAC​CGA​AGA​GCC​ATA​CGA	TCC​TGC​TGC​TGT​TGA​TTC​TGC
Rat-ABCG2	CCA​AAG​CGT​AGT​GTC​TGT​AGC​A	TGC​CCT​GAT​TTA​TCA​AGT​AGT​C
Human-GAPDH	GTG​GAC​CTG​ACC​TGC​CGT​CTA​G	GAG​TGG​GTG​TCG​CTG​TTG​AAG​TC
Human-URAT1	CGC​TTT​GCT​GTT​ACC​CTG​TC	GAG​AGG​CCA​TAG​CTG​AGG​TG
Human-GLUT9	CAG​TGT​TCG​GAT​CCC​TGT​CC	CGA​TAC​TCG​GCT​GCT​CTG​TC
Human-ABCG2	GAA​ACC​TGG​TCT​CAA​CGC​CA	GGT​CGC​GGT​GCT​CCA​TTT​AT

### 2.16 Western blotting analysis

Cells lysis was performed using RIPA buffer (Beyotime, Haimen, China) supplemented with protease and phosphatase inhibitors, and samples were incubated in an ice bath for 1 hour. The cells were subjected to centrifugation at 4°C with a rotational speed of 12,000 rpm, followed by the collection of the supernatant for the extraction of proteins from rat kidney tissue and HK-2 cells. The total protein concentration was quantified using a BCA protein assay kit (TIANGEN, Beijing, China). Protein samples, each containing 30 μg, were loaded onto a 10% SDS-PAGE gel for electrophoretic separation and subsequently transferred onto a PVDF membrane. The membrane was then incubated with 5% skim milk at 37°C for 2 h to block non-specific binding sites. After washing, the PVDF membrane was incubated with the appropriate primary antibody at 4°C overnight. The following primary antibodies were employed to target specific proteins: β-actin (1:5,000), URAT1 (1:1,000), GLUT9 (1:600), ABCG2 (1:1,000), and OAT1 (1:1,000). These antibodies were procured from Wuhan Sanying Biotechnology Co., Ltd., (Wuhan, China). Subsequently, they were incubated with an appropriate secondary antibody conjugated with horseradish peroxidase (HRP), also sourced from Wuhan, China. Protein visualizations were achieved using enhanced chemiluminescence (ECL) and quantitatively analyzed with ImageJ software (Bio-Rad, Hercules, CA, United States). The relative expression levels of the target proteins were calculated using the formula: optical density value of the target protein/optical density value of β-actin.

### 2.17 Statistical analysis

Each experiment was conducted independently and replicated a minimum of three times. The data are presented as the mean ± standard deviation. Statistical analyses were performed using SPSS version 26.0 and GraphPad Prism version 9.0. One-way ANOVA was used among different groups, and Tukey test was used for pairwise comparison analysis. Value of *p* < 0.05 was considered to indicate statistical significance.

## 3 Results

### 3.1 Effects of Wuling capsule on body weight and general indications in HUA rats

**FIGURE 1:**
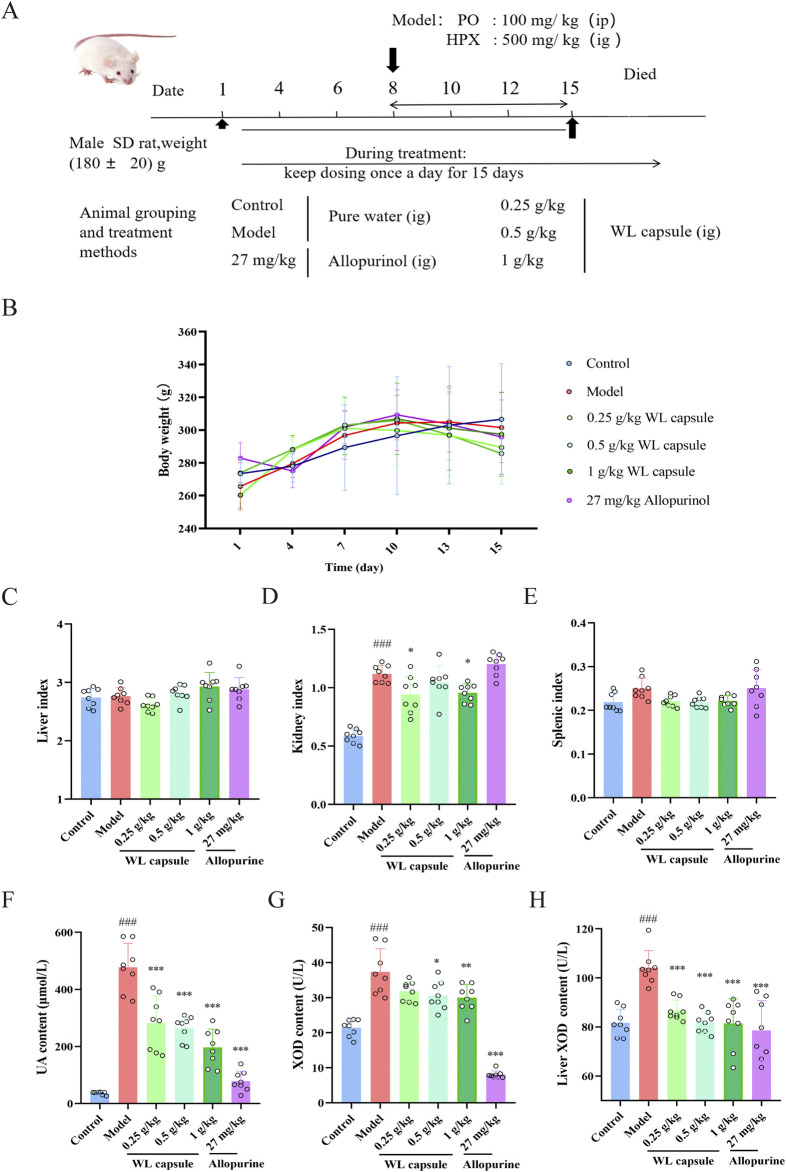
Effects of Wuling Capsule on body weight, organ index, UA and XOD levels in rats. **(A)** Overview of the experimental protocol. **(B)** Alterations in body weight of HUA rats. **(C)** Indices of the liver in rats. **(D)** Indices of the kidney in rats. **(E)** Indices of the spleen in rats. **(F)** Serum UA levels in rats. **(G)** Serum XOD levels in rats. **(H)** Liver XOD expression levels in rats. The data are presented as the means ± standard deviations, n = 8. Compared with the control group, ^###^ *p* < 0.001; compared with the model group, *p < 0.05, **p < 0.01, ***p < 0.001.

The experimental procedure for this study is illustrated in Figure 1A. A rat model of hyperuricaemia was established through the administration of 500 mg/kg hypoxanthine in combination with 100 mg/kg potassium oxonate. Among these six groups, there were no significant differences in body weight (Figure 1B), spleen and liver indexes (Figures 1C, E). However, the kidney index of the model group clearly increased, and it significantly decreased after Wuling capsule treatment (*p* < 0.05) (Figure 1D). The content of UA in serum, as well as the level of XOD in serum and the liver, were markedly increased in HUA rats compared with those in the control group (*p* < 0.001) (Figures 1F–H). These levels were significantly reduced after Wuling capsule treatment (*p* < 0.05). These results showed that Wuling capsule administration could markedly alleviate renal indices, reduce UA content and decrease XOD levels in HUA rats.

### 3.2 Effects of Wuling Capsule on liver histopathology, serum ALT and AST in HUA rats

**FIGURE 2:**
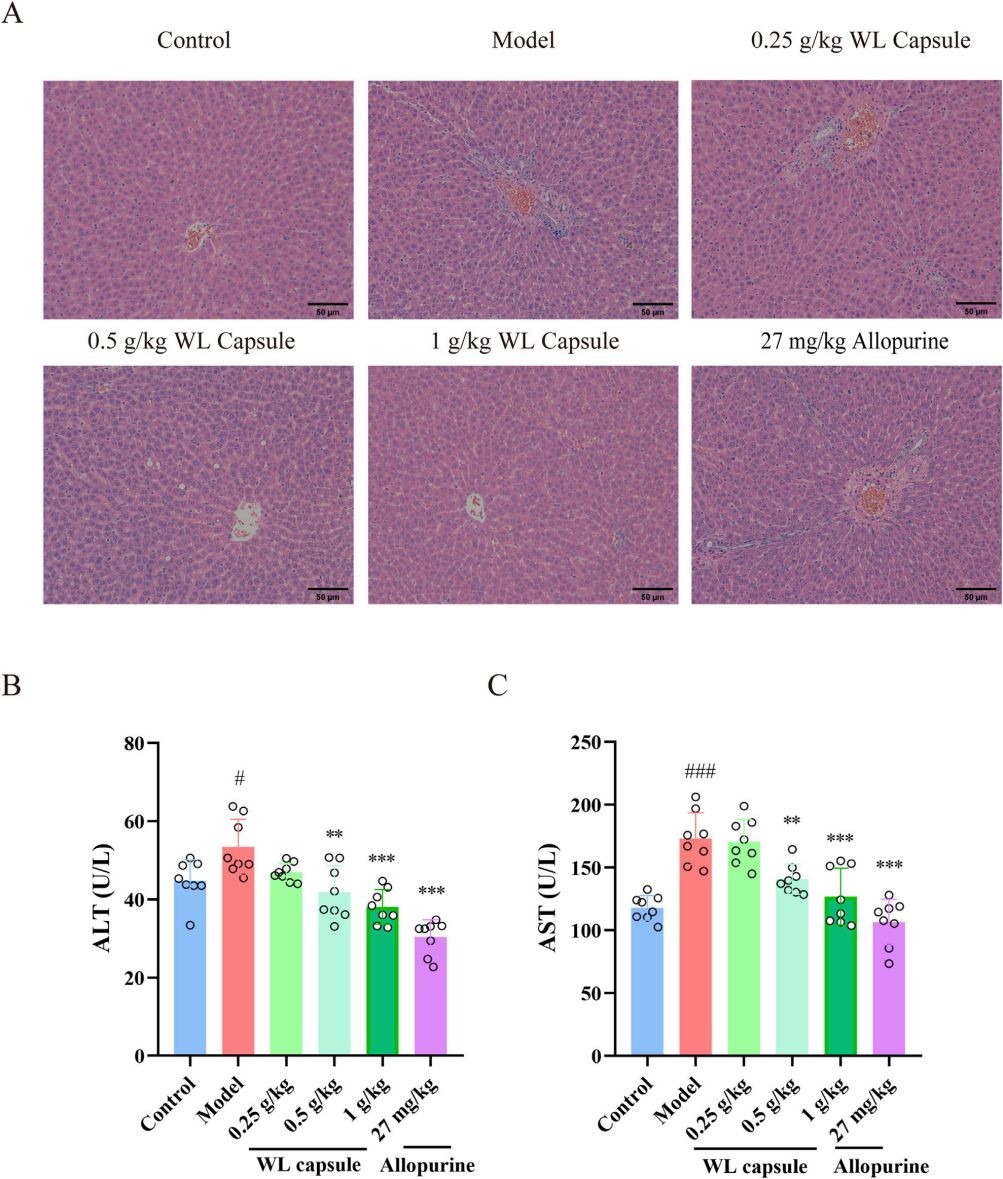
Effects of the Wuling Capsule on liver histopathology, as well as ALT and AST levels in HUA rats. **(A)** Histopathological alterations in the livers of rats. **(B)** The expression levels of ALT in rats. **(C)** The expression levels of AST in rats. The data are presented as the means ± standard deviations, n = 8. Compared with the control group, ^#^ *p* < 0.05, ^###^ *p* < 0.001; compared with the model group, **p < 0.01, ***p < 0.001. The scale length is 50 μm.

Histopathology was performed to investigate the effect of Wuling capsules on liver histopathology of HUA rats. As shown in Figure 2A, the hepatic cells in all groups exhibited a radial arrangement centred on the central vein, and the hepatic lobule structure was preserved with no obvious changes in histopathology. Nevertheless, the levels of ALT and AST were greater in the model group than in the control group, and Wuling capsule treatment reduced the levels of ALT and AST in serum (Figures 2B, C). The results showed that Wuling Capsule had hepatoprotective effects in HUA rats through the inhibition of the serum levels of ALT and AST.

### 3.3 Effects of Wuling Capsule on kidney morphology, histopathology and serum Cr and BUN levels in HUA rats

**FIGURE 3:**
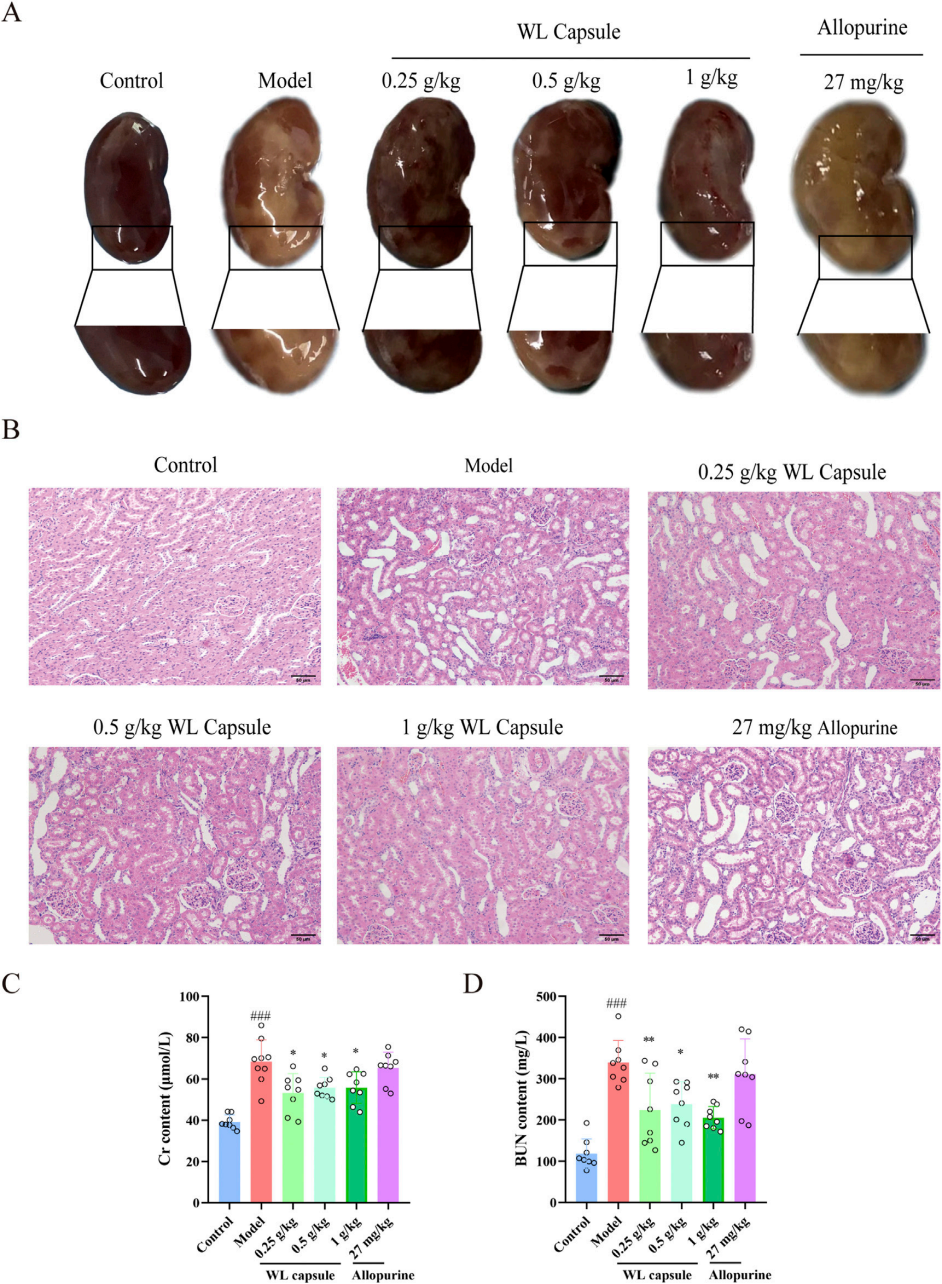
Effects of Wuling Capsule on kidney morphology, histopathology and serum Cr and BUN levels in HUA rats. **(A)** Changes in the morphology of the kidney in HUA rats. **(B)** Changes in kidney histopathology in HUA rats. **(C)** The expression levels of Cr in serum of HUA rats. **(D)** The expression levels of BUN in serum of HUA rats.The data are presented as the means ± standard deviations, n = 8. Compared with the control group, ^###^ *p* < 0.001; compared with the model group, *p < 0.05, **p < 0.01. The scale length is 50 μm.

Compared with those of the normal kidney, the morphology of the kidney in HUA rats revealed numerous yellow plaques on the kidney surface, an obviously enlarged body and reduced elasticity. However, these morphological changes were obviously improved after the administration of Wuling capsule (Figure 3A). HE staining images showed obvious renal tubule dilatation, inflammatory infiltration and cytoplasmic vacuolization in HUA rats. These histopathological changes were obviously alleviated after treatment with Wuling capsules (Figure 3B). In addition, Wuling capsules significantly reduced the production of Cr and BUN in the serum of HUA rats (*p* < 0.05) (Figures 3C, D). The aforementioned results showed the protective effects of Wuling capsule in the kidneys of HUA rats through improving histopathological changes and inhibiting the serum levels of Cr and BUN.

### 3.4 Effects of Wuling capsules on kidney UA-related transporters in HUA rats

**FIGURE 4:**
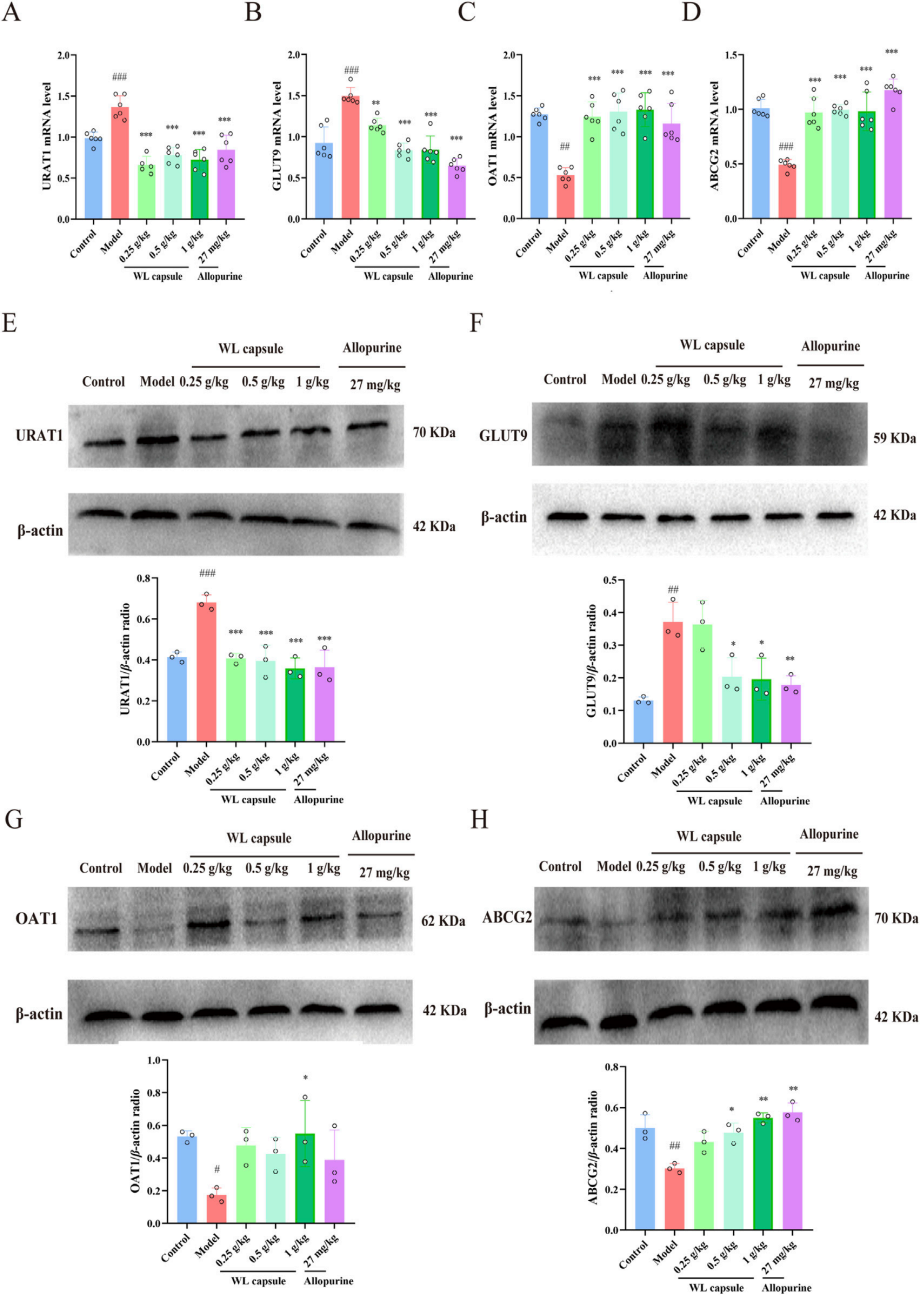
Effect of Wuling Capsule on kidney urate-related transporters in HUA rats. **(A–D)** mRNA expression of URAT1, GLUT9, ABCG2 and OAT1 (n = 6), with GAPDH serving as a blank control. **(E–H)** Protein expression of URAT1, GLUT9, ABCG2 and OAT1 (n = 3), with β-actin used as a blank control. The data are presented as the means ± standard deviations. Compared with the control group, ^#^ *p* < 0.05, ^##^ *p* < 0.01, and ^###^ *p* < 0.001; compared with the model group, *p < 0.05, **p < 0.01, and ***p < 0.001.

As illustrated in Figure 4, the mRNA and protein expression of URAT1 and GLUT9 was markedly upregulated, whereas the mRNA and protein expression of ABCG2 and OAT1 was notably downregulated in the model group compared with that in the control group. Upon supplementation with Wuling capsule, the mRNA and protein expression of ABCG2 (Figures 4D, H) and OAT1 (Figures 4C, G) increased (*p* < 0.05), whereas the expression of URAT1 (Figures 4A, E) and GLUT9 (Figures 4B, F) decreased in the kidney (*p* < 0.05). The results showed that Wuling capsule could modulate the expression of UA-related transporters, specifically URAT1, GLUT9, ABCG2 and OAT1.

### 3.5 Determination of the main constituents from Wuling capsule and its drug-containing serum

**FIGURE 5:**
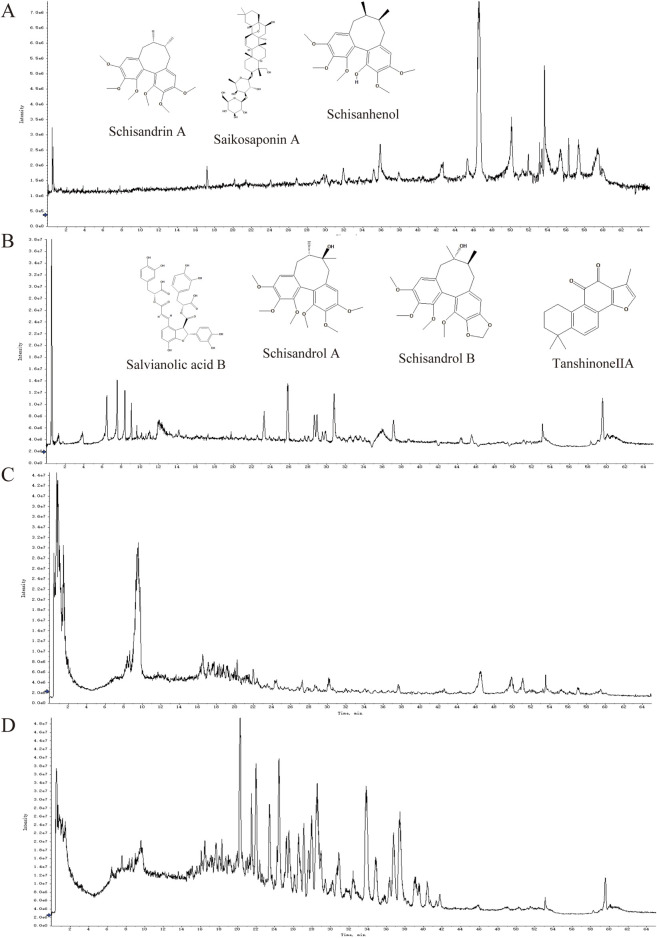
Total ion chromatograms of Wuling Capsule and the serum containing drugs by UPLC-QTOF-MS/MS. **(A)** Serum containing drugs from Wuling capsules in negative mode. **(B)** Serum containing drugs from Wuling Capsules in positive mode. **(C)** Wuling capsules in negative mode. **(D)** Wuling capsules in positive mode.

**FIGURE 6:**
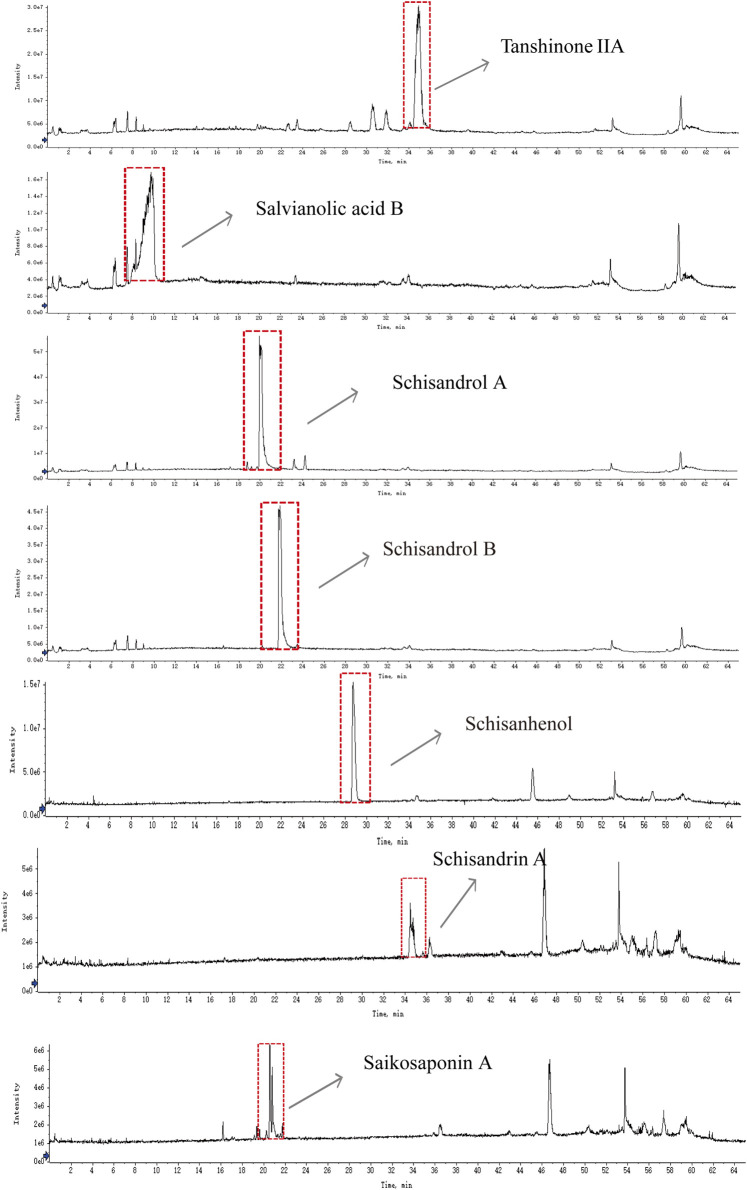
UPLC-Q-TOF-MS/MS chromatograms of tanshinone IIA, salvianolic acid B, schisandrol A, schisandrol B, schisanhenol, Schisandrin A and saikosaponin A.

As shown in Figures 5A–D, forty-one compounds were determined and identified in Wuling capsule based on the relative retention time and evaluation of MS data acquired in both positive and negative ion modes (Supplementary Table S1). Compared with standard substances, seven constituents, including Salvianolic acid B, Schisandrol A, Schisandrol B, Schisanhenol, Schisandrin A, Tanshinone IIA and Saikosaponin A, were present in Wuling capsule and its drug-containing serum (Figure 6). A detailed description of each constituent is provided in Table 2.

**TABLE 2:** The main constituents in the drug-containing serum of Wuling capsules identified by UPLC-Q-TOF/MS/MS.

NO.	Compound	Formula	m/z	Retention time	Ionization model
1	Tanshinone IIA	C_19_H_18_O_3_	295.13	34.87	[M-H]^+^
2	Salvianolic acid B	C_36_H_30_O_16_	718.15	9.86	[M-H]^+^
3	Schisandrol A	C_24_H_32_O_7_	432.21	20.19	[M-H]^+^
4	Schisandrol B	C_23_H_28_O_7_	417.19	21.80	[M-H]^+^
5	Schisanhenol	C_23_H_30_O_6_	402.20	28.51	[M-H]^−^
6	Schisandrin A	C_24_H_32_O_6_	402.03	34.49	[M-H]^−^
7	Saikosaponin A	C_42_H_68_O_13_	826.47	20.46	[M-H]^−^

### 3.6 Effect of the main constituents of Wuling capsule on cell viability in UA-injured HK-2 cells

**FIGURE 7:**
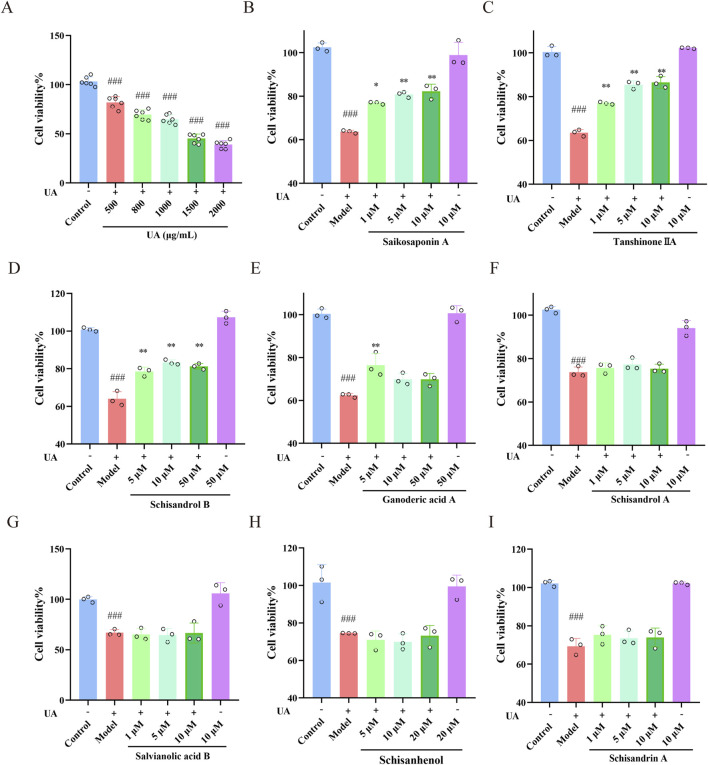
Effects of the main constituents of Wuling capsule on UA-injured HK-2 cells. **(A)** The concentration of UA used to injure HK-2 cells was determined using a CCK-8 assay. **(B–I)** The protective effect of saikosaponin A, tanshinone IIA, schisandrol B, ganoderic acid A, schisandrol A, salvianolic acid B, schisanhenol and schisandrin A on UA-injured HK-2 cells was studied using a CCK-8 assay. The data are presented as the means ± standard deviations; n = 3. Compared with the control group, ^###^ *p* < 0.001; compared with the model group, *p < 0.05, **p < 0.01.

Ganoderic acid A is recognized as a primary constituent of Ganoderma (He et al., 2023), leading us to hypothesize that it may also play a significant role in reducing UA levels and protecting renal function. Therefore, although Ganoderic acid A was not detected in the drug-containing serum of the Wuling capsule in this study, we evaluated the protective effects of the above-mentioned seven constituents, and Ganoderic acid A, on UA-injured HK-2 cells using the CCK-8 assay. The results indicated that these eight constituents exhibited minimal impact on cell viability compared to the control group (Supplementary Figure S2). Subsequently, the effect of varying UA concentrations on cell viability was assessed, identifying 800 μg/mL UA as the optimal concentration for inducing injury in HK-2 cells (Figure 7A). Further analysis demonstrated that Saikosaponin A (10 μM), Tanshinone IIA (5 μM), Schisandrol B (10 μM), and Ganoderic acid A (5 μM) significantly mitigated UA-induced damage in HK-2 cells (Figures 7B–I). These findings underscore the protective effects of Saikosaponin A, Tanshinone IIA, Schisandrol B, and Ganoderic acid A against UA-induced injury in HK-2 cells.

### 3.7 Effects of saikosaponin A, tanshinone IIA, schisandrol B and ganoderic acid A on cell apoptosis in UA-injured HK-2 cells

**FIGURE 8:**
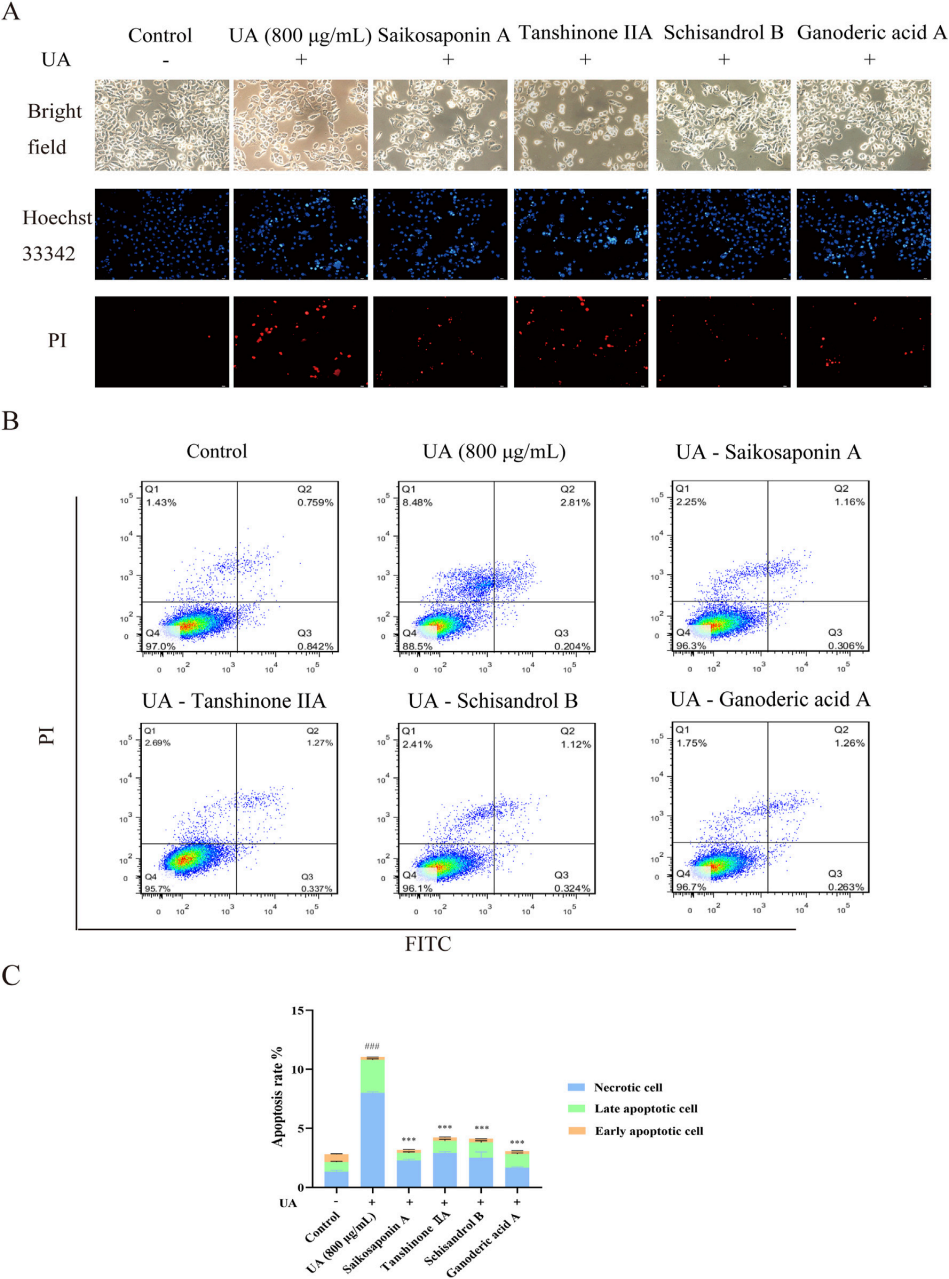
Effects of saikosaponin A, tanshinone IIA, schisandrol B and ganoderic acid A on HK-2 cell apoptosis. **(A)** The nuclear morphology of HK-2 cells (200×) was observed under an inverted fluorescence microscope. **(B,C)** The number of apoptotic cells was detected by flow cytometry. The data are presented as the means ± standard deviations, n = 3. Compared with the control group, ^###^ *p* < 0.001; compared with the model group, ***p < 0.001.

The effects of Saikosaponin A, Tanshinone IIA, Schisandrol B and Ganoderic acid A on the morphology and apoptosis of UA-injured HK-2 cells were determined by Hoechst 33342/PI staining and flow cytometry. As shown in Figure 8A, the main constituents clearly alleviated UA-induced morphological changes (chromatin aggregation and nuclear condensation) and reduced HK-2 cell apoptosis. The percentages of UA-injured HK-2 cells inhibited by Saikosaponin A (10 μM), Tanshinone IIA (5 μM), Schisandrol B (10 μM) and Ganoderic acid A (5 μM) were 28.71%, 38.41%, 37.37%, and 27.83%, respectively (*p* < 0.001) (Figures 8B). These results further demonstrated the protective effects of the four main constituents against UA-induced cell apoptosis.

### 3.8 Binding energy of the main constituents with UA-related transporters was evaluated by molecular docking and molecular dynamics simulation

**FIGURE 9:**
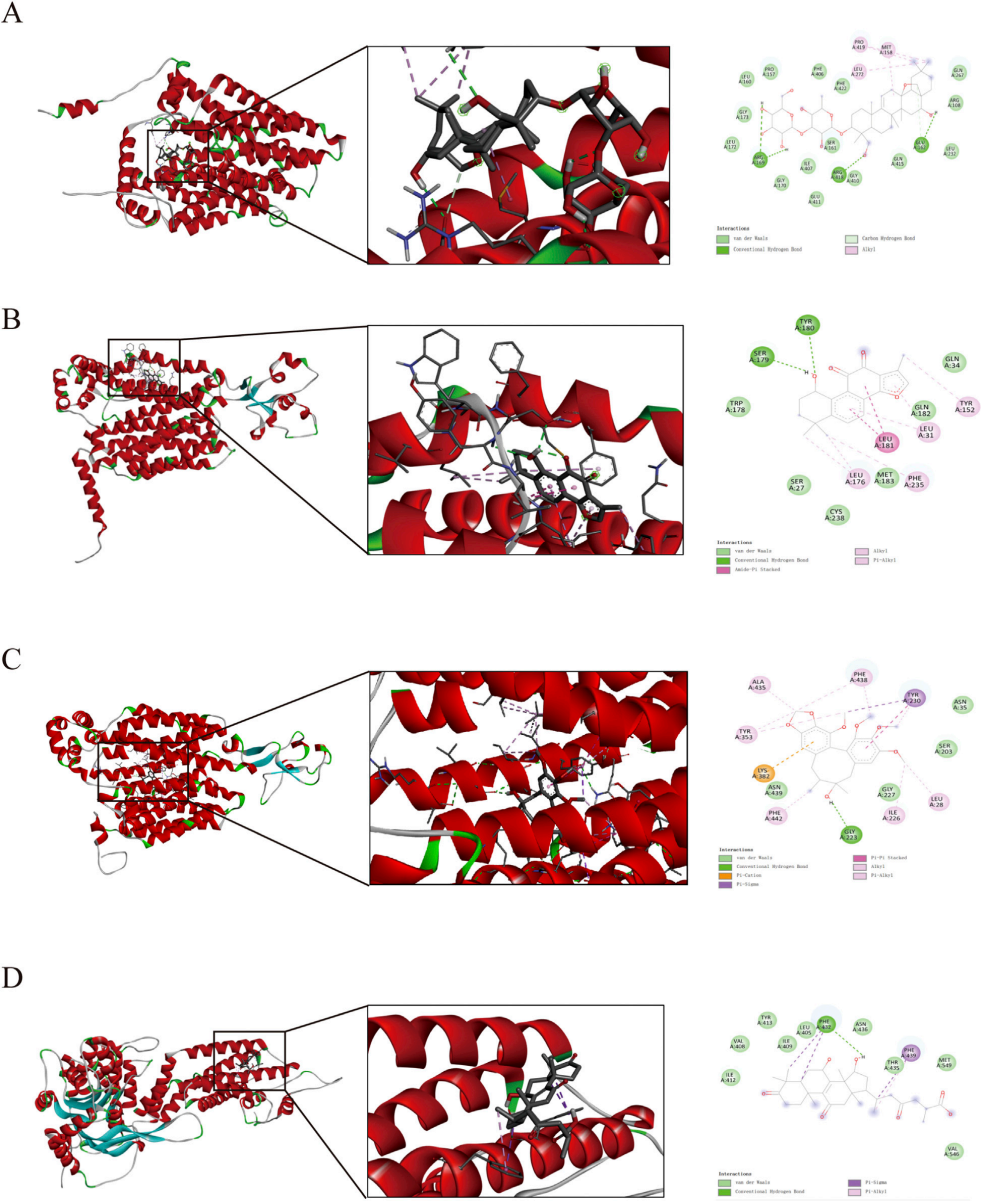
Pattern diagram of molecular docking. **(A)** saikosaponin A and GLUT9. **(B)** tanshinone IIA and URAT1. **(C)** schisandrol B and OAT1. **(D)** ganoderic acid A and ABCG2.

**FIGURE 10:**
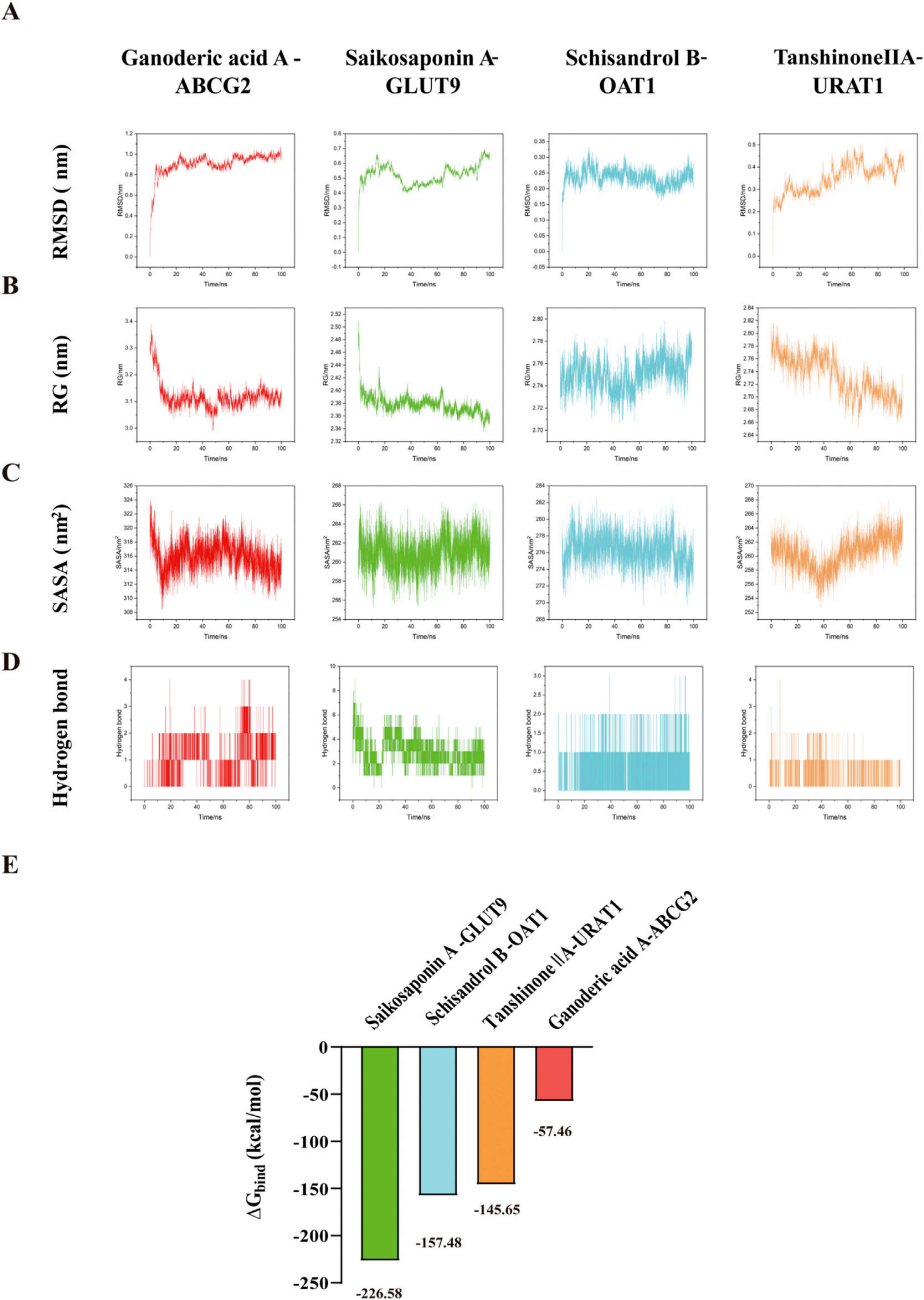
Molecular dynamics simulation and binding free energy. **(A)** RMSD. **(B)** RG. **(C)** SASA. **(D)** Hydrogen bond. **(E)** Binding free energies (∆G_bind_).

In further work, the conformational rationality of UA-related transporters was evaluated (Supplementary Figure S1). The binding mechanisms are shown in Supplementary Figure S3, and the binding energies are shown in Supplementary Table S2. Notably, Saikosaponin A, Tanshinone IIA, Schisandrol B, and Ganoderic acid A, along with their selected UA-related transporters, exhibit strong binding energies, as shown in Figures 9A–D. The stability of the binding energy between constituents and key targets can be verified by molecular dynamics simulations (Sarker et al., 2023). As shown in Figure 10A, the four complexes quickly stabilized at 0.91 ± 0.11 nm, 0.52 ± 0.07 nm, 0.24 ± 0.03 nm and 0.24 ± 0.26 nm. During the simulation, the Rg values of the complex were stable at 3.12 ± 0.05 nm, 2.38 ± 0.02 nm, 2.75 ± 0.01 nm, and 2.73 ± 0.03 nm (Figure 10B). The SASA values of the four complexes were stable at 315.91 ± 2.15 nm^2^, 260.99 ± 1.71 nm^2^, 276.28 ± 1.80 nm^2^ and 260.49 ± 2.28 nm^2^ respectively (Figure 10C). The hydrogen bond density and strength of the four complexes were ordered as follows: Saikosaponin A-GLUT9, Schisandrol B-OAT1, Tanshinone IIA-URAT1 and Ganoderic acid A-ABCG2 (Figure 10D). The binding free energy (∆G_bind_) of these complexes was obtained using MM/PBSA method. A lower value of ∆G_bind_ indicates a stronger binding affinity between the receptor and the ligand (Marciniak et al., 2022). As shown in Figure 10E, the ∆G_bind_ (kcal/mol) for the four complexes is shown as follows: Saikosaponin A-GLUT9 (−226.58 kcal/mol), Schisandrol B-OAT1 (−157.48 kcal/mol), Tanshinone IIA-URAT1 (−145.65 kcal/mol), Ganoderic acid A-ABCG2 (−57.46 kcal/mol). These results demonstrated the four main constituents and the indicated UA-related transporters exhibit strong and stable binding energy.

### 3.9 Effects of saikosaponin A, tanshinone IIA, schisandrol B and ganoderic acid A on the mRNA and protein expression of URAT1, GLUT9 and ABCG2 in UA-injured HK-2 cells

**FIGURE 11:**
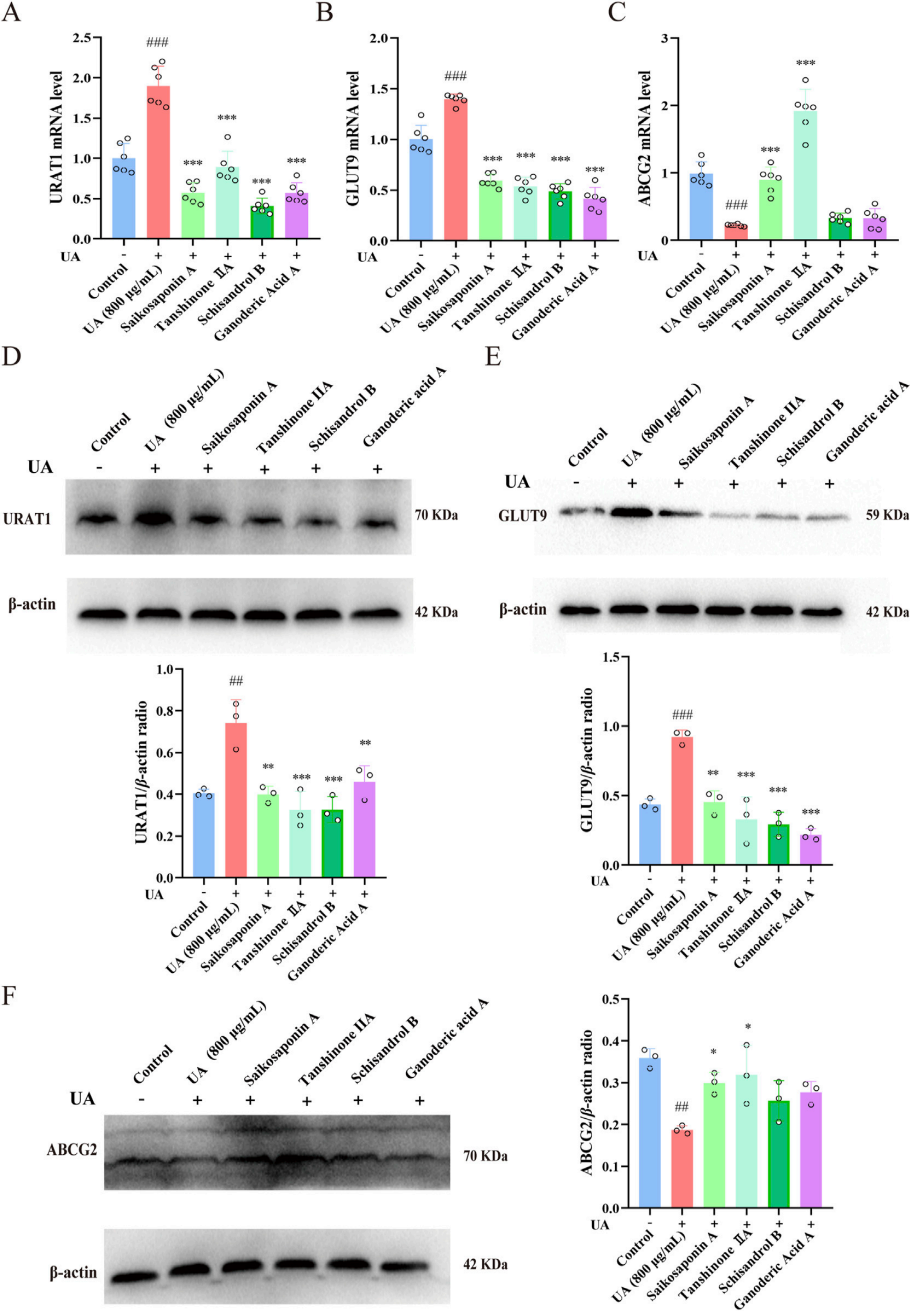
Effects of saikosaponin A, tanshinone IIA, schisandrol B and ganoderic acid A on URAT1, GLUT9 and ABCG2 mRNA and protein expression in UA-injured HK-2 cells. **(A–C)** Expression of URAT1, GLUT9 and ABCG2 mRNA (n = 6), with GAPDH serving as a blank control. **(D–F)** Expression of the URAT1, GLUT9 and ABCG2 proteins (n = 3), with β-actin used as a blank control. The data are presented as the means ± standard deviations. Compared with the control group, ^###^ *p* < 0.001 and ^##^ *p* < 0.01; compared with the model group, *p < 0.05, **p < 0.01, and ***p < 0.001.

As shown in Figures 11A–F, the mRNA and protein expression of URAT1 and GLUT9 were markedly upregulated, whereas the ABCG2 mRNA and protein levels were markedly downregulated in UA-injured HK-2 cells compared with those in control cells (*p* < 0.01). Nevertheless, when administered, Saikosaponin A and Tanshinone IIA decreased the expression of URAT1 and GLUT9 and increased the expression of ABCG2 in HK-2 cells (*p* < 0.05). However, Schisandrol B (10 μM) and Ganoderic acid A (5 μM) decreased the expression of URAT1 and GLUT9 but had little effect on the expression of ABCG2. These results demonstrated that the protective effects of these constituents on UA-injured HK-2 cells occurred mainly through the modulation of the expression of the UA-related transporters URAT1, GLUT9 and ABCG2.

## 4 Discussion

**FIGURE 12:**
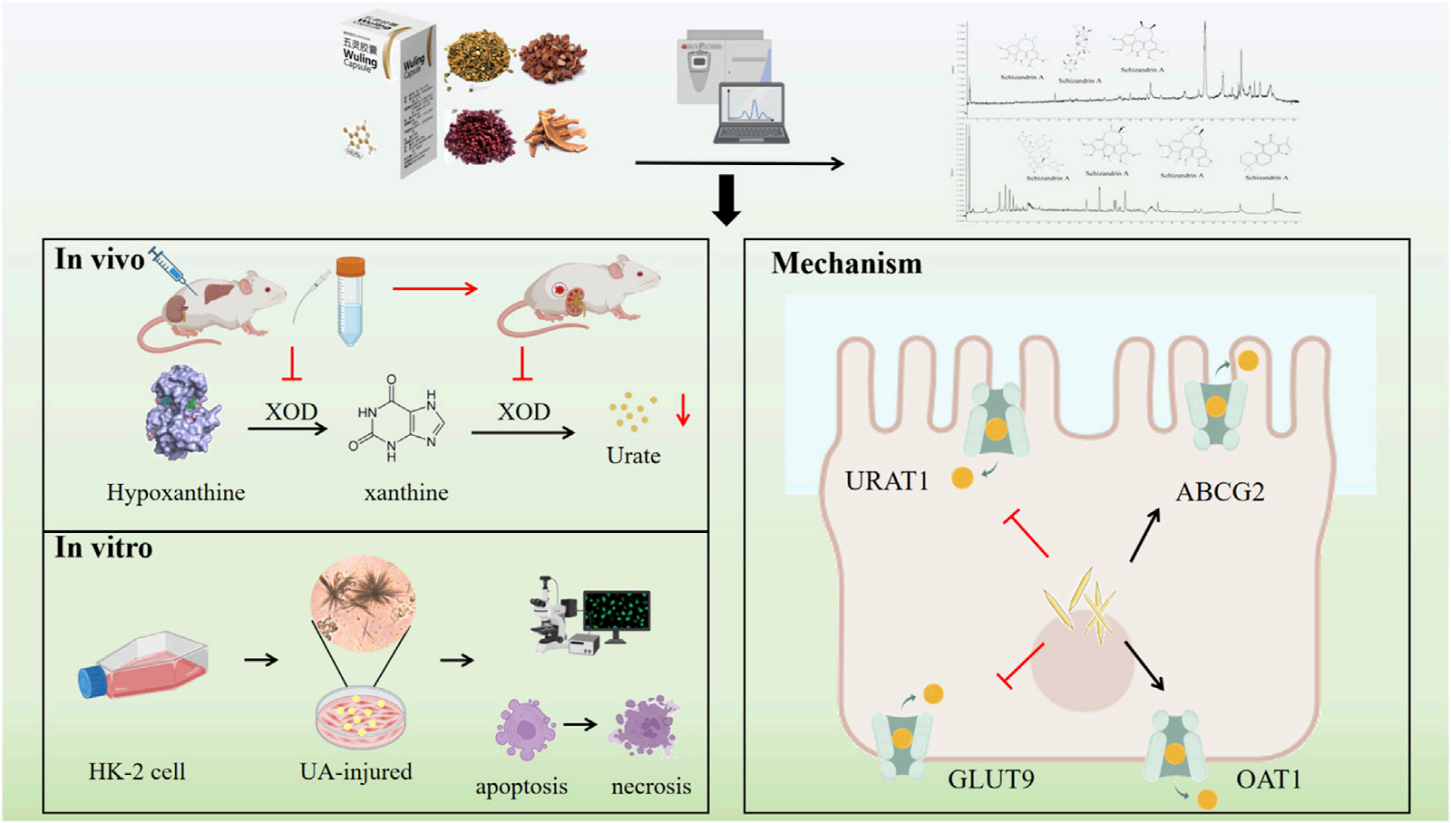
Mechanisms underlying the anti-hyperuricaemia effects of Wuling capsule.

Hyperuricaemia, a chronic metabolic disorder, results from abnormalities in purine metabolism, culminating in increased concentrations of UA in the bloodstream. These elevated UA levels can precipitate the formation of MSU crystals, which are implicated in the inflammatory processes characteristic of gout and other associated conditions. Moreover, previous study demonstrated that individuals with hyperuricemia frequently exhibit hepatic and renal insufficiencies, with over 50% of these patients also presenting with fatty liver disease (Yu et al., 2023). Within the liver, elevated levels of UA contribute to hepatocellular damage by promoting insulin resistance and oxidative stress, which in turn stimulate the release of inflammatory mediators and other pathways, thereby further aggravating liver damage (Wang et al., 2025). Research has demonstrated that certain agents can effectively decrease UA production to mitigate liver damage associated with hyperuricemia through regulating the oxidative stress levels and the NLRP3 pathway (Ai et al., 2023). Kang et al. conducted a study that revealed significant associations between up to 123 lipids and UA production, with a predominant focus on glycerides and glycerophospholipids (Kang et al., 2024). These studies highlight that mitigate UA-induced liver damage is significant meaningful for HUA treatment. In addition to liver, the kidney also acts as a key regulator for UA levels, which ensures that approximately 66% of daily UA is cleared through urine. Supersaturated UA in human blood precipitates UA crystals, which are deposited in renal tubules and the renal interstitium, causing chronic local inflammation, immune damage and microvascular lesions of the kidney (Yang et al., 2024). Therefore, improving liver and kidney function, reducing UA synthesis and promoting UA excretion could be effective strategies for the treatment of HUA (Liu et al., 2023). The hepatoprotective efficacy of the Wuling capsule has been substantiated through both experimental and clinical investigations (Committee, 2020; Ren et al., 2024). Additionally, we have conducted preliminary elucidations of its anti-gout properties, which are associated with the modulation of the TLR4/NF-κB signaling pathway (Li et al., 2024). In this study, we developed a HUA rat model using hypoxanthine combined with potassium oxonate, alongside a UA-injured HK-2 cell model, to examine the therapeutic effects and underlying mechanisms of the Wuling capsule *in vivo* and *in vitro* (Figure 12).

Hypoxanthine supplementation simulates the normal purine metabolic pathway in humans, representing a critical step in UA production. Potassium oxonate acts as an inhibitor of xanthine oxidase, suppressing UA excretion. When used in combination with hypoxanthine, potassium oxonate mimics the authentic pathogenesis of human hyperuricemia patients from both “increased production” and “decreased excretion” perspectives. This work employed a HUA rat model induced by hypoxanthine combined with potassium oxonate. The serum UA levels increased to about 500 μmol/L in HUA rats, which is consistent with the previous works, indicating that the model in this study is successful (Xu et al., 2021). A reduction in the serum UA level is the most intuitive indicator of a UA lowering effect, and Wuling capsule could significantly reduce the serum UA level in HUA rats, indicating that Wuling capsule has a lowering effect on the UA level. The occurrence of HUA is related to the anabolism of UA in the liver (Sun et al., 2021). XOD is a key enzyme for the metabolism of purine nucleotides to UA, which has long been considered a key target in the development of UA-lowering drugs. Hypoxanthine is oxidized to xanthine under the action of XOD, and xanthine is further oxidized to UA (Liang et al., 2023). Wuling capsule could obviously reduce the serum and liver XOD level in HUA rats, suggesting that the lowering UA effect was associated with XOD regulation. As is well known that ALT and AST are the most commonly used and sensitive indicators of liver function in clinic (Chinnappan et al., 2023). In this work, although there were no obvious changes in the HE-stained images of the liver among all groups, ALT and AST in the serum were overproduced in HUA rats, indicating that the liver had suffered certain damage. However, Wuling capsule could reduce the expression of ALT and AST in HUA rats. These findings suggest that Wuling capsule could treat HUA by reducing UA, inhibiting XOD and protecting liver function.

Sreum Cr and BUN are considered as pivotal clinical indicators for assessing kidney function and injury. These biomarkers provide insights into the kidney’s ability to filter waste products from the blood. In HUA rats, Cr and BUN levels were usually monitored to assess early renal injury (Xu et al., 2019). This study obviously observed renal tubule dilation and vacuolation of renal tubule epithelial cells in HUA rats, suggesting that kidney damage was caused directly or indirectly by increased UA levels, eventually triggering an increase in Cr and BUN levels. A significant aspect of this study is the observation that, although allopurinol effectively reduced blood UA levels in HUA rats, it simultaneously caused renal damage. This was evidenced by elevated levels of Cr, BUN as well as renal tubular dilation and interstitial edema in the kidneys, compared to the control group (Wei et al., 2022). The finding is consistent with the previous studies (Shu et al., 2022). Notably, Wuling capsule administration not only improved HE staining images and alleviated the dilation of renal tubules in HUA rats, but also effectively decreased the content of Cr and BUN in the blood. These results suggested that Wuling capsule could be used to treat HUA by improving kidney function.

HK-2 cells are derived from human proximal renal tubular epithelial cells. *In vivo*, when UA induces kidney damage, the proximal renal tubules are the primary target site. The UA-induced HK-2 cell injury model accurately replicates the fundamental pathological processes associated with hyperuricemia-induced renal lesions (Liu et al., 2022). This model enables a comprehensive investigation of the pathogenic mechanisms of UA at the renal cellular level. In order to better evaluate the effect of Wuling Capsule *in vitro*, the main constituents of Wuling Capsule and its drug-containing serum were determined. Forty-one compounds were determined and identified in Wuling Capsule. Seven constituents, including Salvianolic acid B, Schisandrol A, Schisandrol B, Schisanhenol, Schisandrin A, Tanshinone IIA and Saikosaponin A, were present in both Wuling capsule and its drug-containing serum. Actually, there are six components match the nine components determined in Wuling capsule in our previous work (Ren et al., 2024). Nevertheless, Schisanhenol was only found in this work. In addition, we infer that Ganoderic acid A might also be one of the main component of Wuling capsule for protecting kidney. Therefore, the protection effect of total of eight constituents was investigated *in vitro*. As expected, the eight compounds had little cytotoxicity on cell viability of HK-2 cells. Among them, Saikosaponin A, Tanshinone IIA, Schisandrol B and Ganoderic acid A had better protective effects on UA-injured HK-2 cells. Moreover, these four constituents exhibited a strong protective effect on UA-induced cell apoptosis. Specifically, the renoprotection effects of Tanshinone IIA and Ganoderic acid A were consistent with the previous works (Geng et al., 2020; Zhang et al., 2024), and we provided the first experimental evidences that the renoprotection effect of Saikosaponin A and Schisandrol B in UA-induced injury in HK-2 cells. These findings indicated that Saikosaponin A, Tanshinone IIA, Schisandrol B and Ganoderic acid A in Wuling capsules might protect HK-2 cells from UA injury by reducing cell apoptosis.

While the mechanism of UA reduction is linked to various factors, including the inhibition of XOD activity, anti-inflammatory and antioxidant effects, the interaction of transport regulation mechanisms holds a pivotal role (Du et al., 2024). Studies have shown that high expression of URAT1 and GLUT9 in the kidney can transport UA from the renal interstitium to renal tubular epithelial cells and then back into the serum thereby increasing the serum UA level (Kim and June, 2022). Researchers observed a significant increase in serum UA levels in mice following the knockout of the OAT1 gene, underscoring the essential role of organic anion transporters in the pathogenesis of hyperuricemia (Lowenstein and Nigam, 2021). Another study employing ABCG2 knockout mouse models have demonstrated that ABCG2 dysfunction leads to reduced extrarenal urate excretion, consequently inducing hyperuricemia (Chi et al., 2024). *In vitro* experiments involving the heterologous expression of ABCG2 have confirmed that, in the presence of adenosine triphosphate (ATP), ABCG2 mediates urate transport (Ristic et al., 2020). Currently, multiple pharmacological agents are designed to target uric acid transporters. For instances, Salinomycin, a robust inhibitor of XOD and URAT1, mitigates hyperuricemic nephropathy through the activation of NRF2, modulation of gut microbiota, and enhancement of short-chain fatty acid (SCFA) production (Chen et al., 2024). Lesinurad exhibits a dual inhibitory action on URAT1 and GLUT9, significantly impeding urate reabsorption (Shi et al., 2024). Those evidences strongly support that inhibition of URAT1 and GLUT9 expression, as well as promotion of ABCG2 and OAT1 expression, are important strategies to accelerate UA excretion (Liu et al., 2024). This study investigated whether the mechanism by which Wuling capsule reduces UA levels involves the regulation of UA transports. Molecular docking and molecular dynamics simulation predicted that the above four constituents in Wuling capsule had strong binding energies with the four UA-related transporters. Moreover, Wuling capsule significantly decreased the expression of URAT1 and GLUT9 and promoted the expression of ABCG2 and OAT1 in kidney tissue of HUA rats and in UA-injured HK-2 cells. These results indicated that Wuling capsule could treat HUA by regulating the expression of these transporters. Nevertheless, this study has several limitations. First, the UA-lowering effect of Wuling capsule was assessed using a model of hyperuricemia induced by hypoxanthine and potassium oxonate in rats; future research should employ alternative modeling methods. Second, the formation and excretion of UA are intricately linked to the liver kidney and intestine each of which plays a crucial role in maintaining UA homeostasis. The effects of Wuling capsule on the intestinal barrier and microbiota remain unclear. Third, further investigation is needed to identify additional constituents of Wuling capsule and to elucidate the molecular mechanisms underlying its UA-lowering effects.

## 5 Conclusion

This study demonstrated the therapeutic effect of Wuling capsule on HUA both *in vivo* and *in vitro*, primarily by protecting liver and kidney function, improving the regulation of cell apoptosis, and regulating UA-related transporters in the kidney to reduce serum UA levels. The main bioactive constituents were Saikosaponin A, Tanshinone IIA, Schisandrol B and Ganoderic acid A. This study elucidated the effects and mechanisms of Wuling capsule in treating HUA, and further provided a scientific basis for Wuling capsule in new clinical applications.

## Data Availability

The raw data supporting the conclusions of this article will be made available by the authors, without undue reservation.

## Abbreviations

MSU, mono-sodium urate; AGA, acute gouty arthritis; UA, uric acid; HUA, hyperuricaemia; HE, haematoxylin-eosin; XOD, xanthine oxidase; ALT, alanine Aminotransferase; AST, aspartate transaminase; Cr, creatinine; BUN, blood urea nitrogen; URAT1, urate transporter 1; GLUT9, Glucose Transporter 9; OAT1, Organic Anion Transporter 1; ABCG2, ATP-binding cassette subfamily G member 2; HK-2 cells, human kidney 2 cells.
