# Supplementary material for: Wuling capsule alleviates hyperuricaemia and protects UA- injured HK-2 cells by regulating uric acid transporter proteins
Source: Front Pharmacol. 2025 Apr 29;16:1563676. doi: 10.3389/fphar.2025.1563676 (PMC12069045; doi:10.3389/fphar.2025.1563676)
Supplement: Supplementary file 1 [file DataSheet1.docx]

Supplementary Material

Wuling capsule alleviates hyperuricaemia and protects UA- injured HK-2 cells by regulating uric acid transporter proteins

**Nan Li, Hongna Liu, Zhongxing Song, Rui Zhou ^*^, Zhishu Tang^*^, Hongbo Xu, Xinbo Shi, Yanru Liu, Jian Ni**

*** Correspondence:**

Rui Zhou: [zhouruiruiswg@126.com](mailto:zhouruiruiswg@126.com) ;

Zhishu Tang: [tzs6565@163.com](mailto:tzs6565@163.com)

**Supplementary Data**

**Table S1. Wuling capsule and drug-containing serum constituents**

**Table S2. Binding energy of the four main constituents and four UA-related transporters.**

**Figure S1. The homology models of in human from SWISS-MODEL.**

**Figure S2. Effects of main components of Wuling capsule on HK-2 cells.**

**Figure S3. The molecular docking results of the four main constituents and four UA-related transporters.**

Table S1. Wuling capsule and drug-containing serum constituents

| NO. | Compound | Formula | m/z | Retention time | Medicated serum（√） |
| --- | --- | --- | --- | --- | --- |
| 1 | Sodium ferulate | C_10_H_9_NaO_4_ | 216.04 | 0.46 | √ |
| 2 | Citric acid | C_6_H_8_O_7_ | 192.03 | 0.51 | √ |
| 3 | Tanshinone I | C_18_H_12_O_3_ | 276.08 | 0.53 | √ |
| 4 | Glabridin | C_20_H_20_O_4_ | 324.14 | 1.27 | √ |
| 5 | Alvianolic acid G | C_18_H_12_O_7_ | 341.07 | 8.31 | - |
| 6 | Schisandrin A | C_24_H_32_O_6_ | 416.22 | 8.46 | √ |
| 7 | HeteroclitinD | C_27_H_30_O_8_ | 482.19 | 10.93 | √ |
| 8 | Aikochromoside A | C_17_H_20_O_10_ | 385.11 | 11.00 | - |
| 9 | Anshinol A | C_18_H_12_O_4_ | 293.08 | 12.15 | - |
| 10 | p-Aminocinnamic acid | C_9_H_9_NO_2_ | 163.06 | 12.16 | √ |
| 11 | Rosmarinic acid | C_18_H_16_O_8_ | 360.08 | 13.01 | √ |
| 12 | Cinnamaldehyde | C_9_H_8_O | 132.06 | 13.16 | √ |
| 13 | Salvianolic Acid A | C_26_H_22_O_10_ | 494.12 | 13.42 | √ |
| 14 | Salvianolic Acid B | C_36_H_30_O_16_ | 718.15 | 14.10 | √ |
| 15 | Schizandrol B | C_23_H_28_O_7_ | 416.18 | 14.93 | √ |
| 16 | Sativin | C_17_H_18_O_4_ | 286.12 | 17.01 | √ |
| 17 | Uridine | C_9_H_12_N_2_O_6_ | 244.07 | 19.28 | √ |
| 18 | Ganoderic acid A | C_30_H_44_O_7_ | 516.31 | 19.41 | √ |
| 19 | Schisandrin | C_24_H_32_O_7_ | 432.21 | 20.13 | √ |
| 20 | Saikosaponin A | C_42_H_68_O_13_ | 826.47 | 21.36 | √ |
| 21 | Schisandrin | C_24_H_32_O_7_ | 432.21 | 22.41 | √ |
| 22 | Schisanlignone A | C_24_H_30_O_7_ | 430.20 | 24.05 | √ |
| 23 | Cryptotanshinone | C_19_H_20_O_3_ | 296.14 | 25.59 | √ |
| 24 | Schisantherin A | C_30_H_32_O_9_ | 536.20 | 28.08 | √ |
| 25 | Ganoderic acid I | C_30_H_44_O_8_ | 532.30 | 31.90 | √ |
| 26 | All-trans-Retinoic acid | C_20_H_28_O_2_ | 300.21 | 33.52 | √ |
| 27 | Tanshinone IIA | C_19_H_18_O_3_ | 294.13 | 34.30 | √ |
| 28 | Sanguiin H1 | C_34_H_26_O_22_ | 786.09 | 35.89 | √ |
| 29 | Schisandrin B | C_23_H_28_O_6_ | 400.19 | 36.88 | √ |
| 30 | Schizandrin C | C_22_H_24_O_6_ | 384.16 | 39.57 | - |
| 31 | Ethyllaurate | C_14_H_28_O_2_ | 228.21 | 42.49 | √ |
| 32 | Geraniin | C_41_H_28_O_27_ | 975.07 | 42.67 | √ |
| 33 | Sylvatine | C_24_H_33_NO_3_ | 383.25 | 46.54 | √ |
| 34 | Schisanhenol | C_23_H_30_O_6_ | 402.20 | 46.56 | √ |
| 35 | Linoleic acid | C_18_H_32_O_2_ | 280.24 | 46.73 | √ |
| 36 | Taxinine | C_35_H_42_O_9_ | 606.28 | 52.89 | √ |
| 37 | Stearic acid | C_18_H_36_O_2_ | 284.27 | 53.37 | √ |
| 38 | Schisanlactone B | C_30_H_42_O_4_ | 466.31 | 53.41 | √ |
| 39 | Tangeretin | C_20_H_20_O_7_ | 372.12 | 53.64 | √ |
| 40 | Ganoderic acid E | C_30_H_40_O_7_ | 512.28 | 55.53 | √ |
| 41 | Fraxin | C_16_H_18_O_10_ | 370.09 | 56.84 | √ |

Table S2. Binding energy of the four main constituents and four UA-related transporters.

|  | **URAT1** | **GLUT9** | **ABCG2** | **OAT1** |
| --- | --- | --- | --- | --- |
| Saikosaponin A | -1.41 | -4.18 | -3.43 | -2.91 |
| TanshinoneⅡA | -7.96 | -7.82 | -6.18 | -6.68 |
| Schisandrol B | -5.25 | -5.3 | -4.33 | -5.06 |
| Ganoderic acid A | -5.2 | -7.38 | -4.98 | 2.56 |


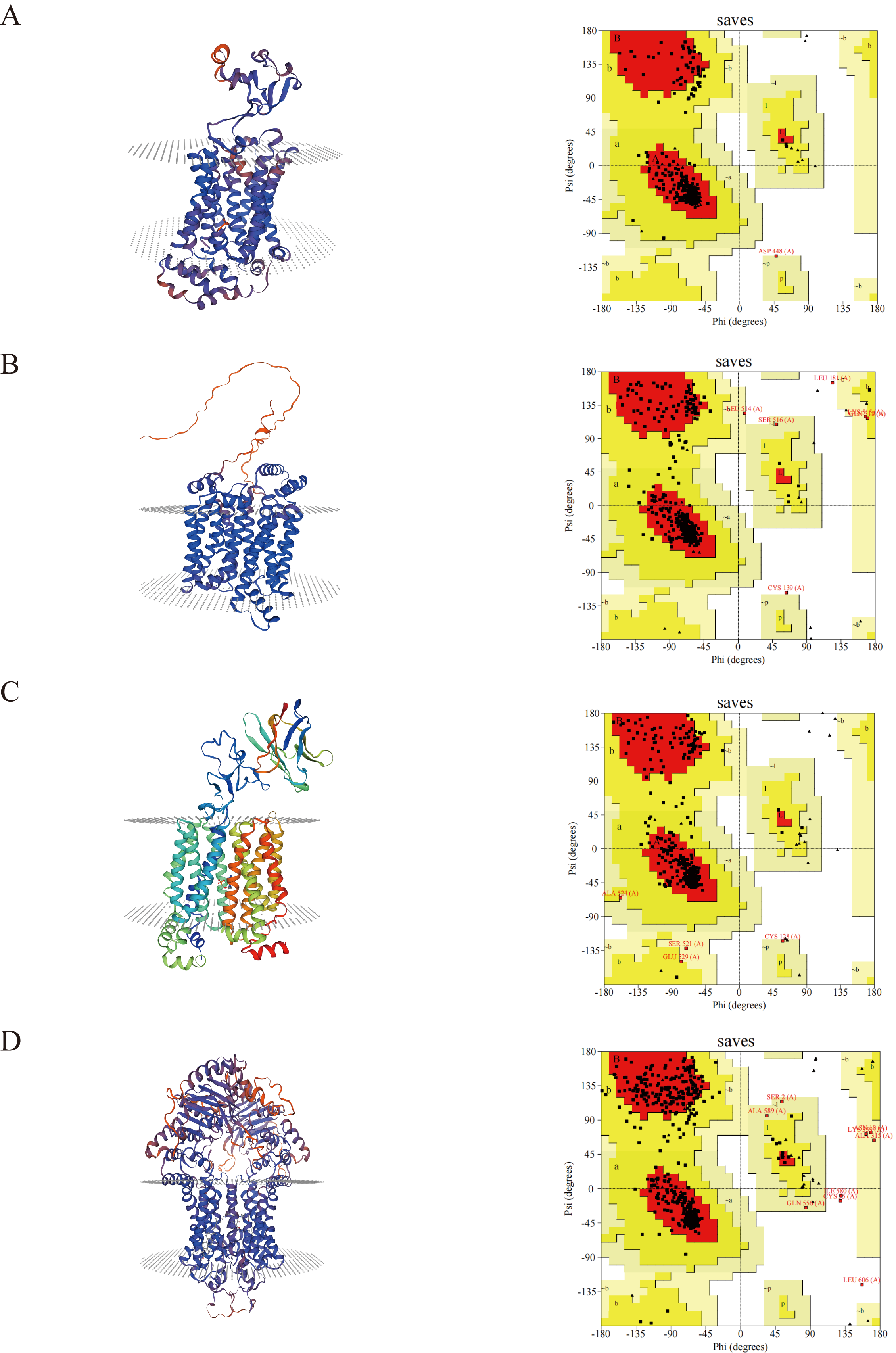


Fig. S1. The homology models of in human from SWISS-MODEL. **(**A) URTA1. (B) GLUT9. (C) OAT1. (D) ABCG2.


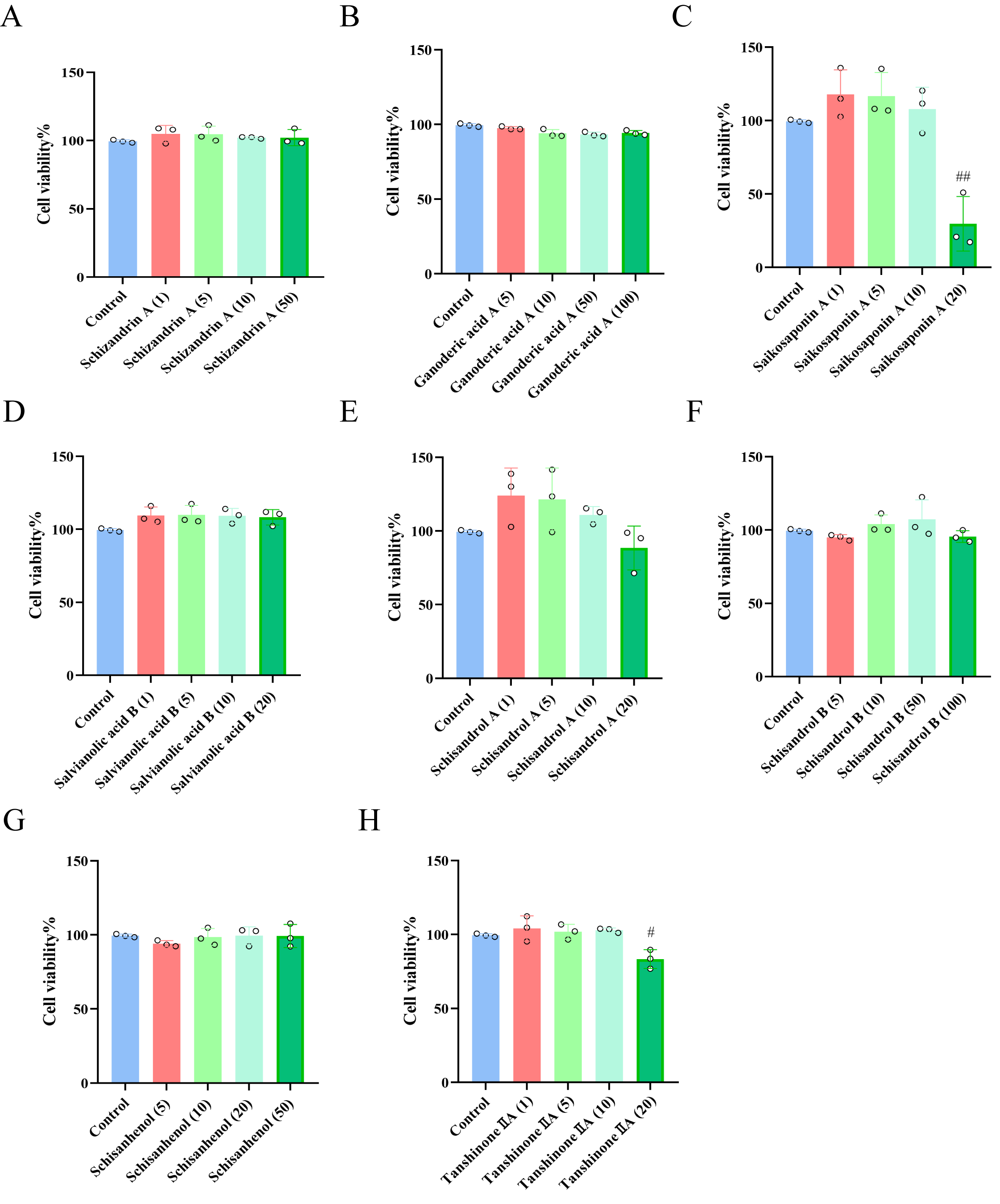


Fig. S2. Effects of main components of Wuling capsule on HK-2 cells. (A) Schisandrin A. (B) Ganoderic acid A. (C) Saikosaponin A. (D) Salvianolic acid B. (E) Schisandrol A. (F) Schisandrol B. (G) Schisanhenol. (H) Tanshinone IIA.


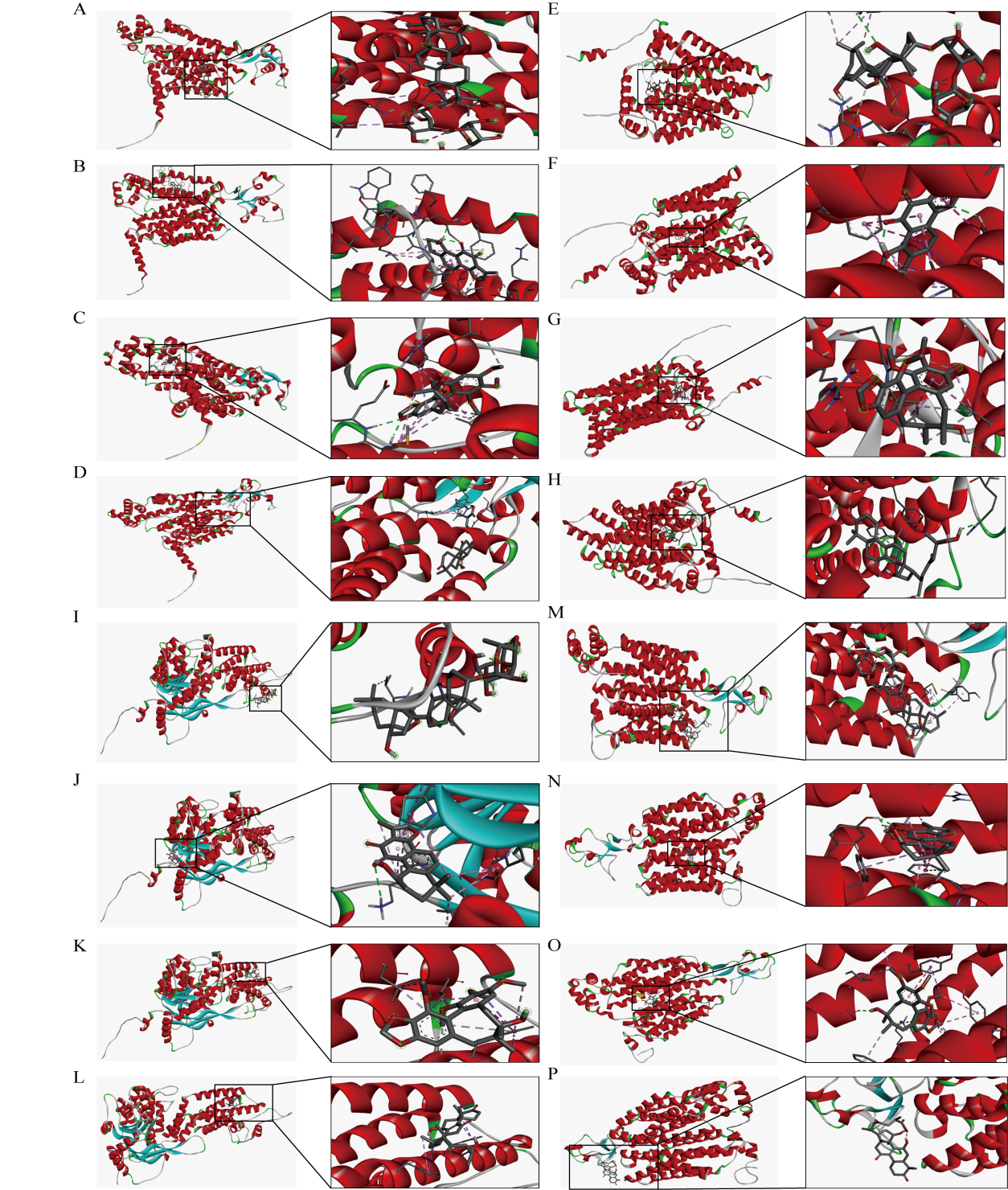


Fig. S3. The molecular docking results of the four main constituents and four UA-related transporters. (A) Saikosaponin A and URAT1. (B) TanshinoneⅡA and URAT1. (C) Schisandrol B and URAT1. (D) Ganoderic acid A and URAT1. (E) Saikosaponin A and GLUT9. (F) TanshinoneⅡA and GLUT9. (G) Schisandrol B and GLUT9. (H) Ganoderic acid A and GLUT9. (I) Saikosaponin A and ABCG2. (J) TanshinoneⅡA and ABCG2. (K) Schisandrol B and ABCG2. (L) Ganoderic acid A and ABCG2. (M) Saikosaponin A and OAT1. (N) TanshinoneⅡA and OAT1. (O) Schisandrol B and OAT1. (P) Ganoderic acid A and OAT1.
